# Associations between snoring, body mass index, and coronary artery diseases: Observational and Mendelian randomization study in Asia Snoring, BMI, and CAD among East Asians

**DOI:** 10.1111/resp.14893

**Published:** 2025-02-05

**Authors:** Yunqing Zhu, Yongbing Lan, Jun Lv, Dianjianyi Sun, Pei Pei, Ling Yang, Iona Y. Millwood, Robin G.Walters, Yiping Chen, Huaidong Du, Jian Wang, Xiaoming Yang, Rebecca Stevens, Junshi Chen, Zhengming Chen, Liming Li, Canqing Yu

**Affiliations:** a Department of Epidemiology & Biostatistics, School of Public Health, Peking University, Beijing 100191, China; b Peking University Center for Public Health and Epidemic Preparedness & Response, Beijing 100191, China; c Key Laboratory of Epidemiology of Major Diseases ( Peking University), Ministry of Education, Beijing 100191, China; d Medical Research Council Population Health Research Unit at the University of Oxford, Oxford OX3 7LF, United Kingdom; e Clinical Trial Service Unit & Epidemiological Studies Unit (CTSU), Nuffield Department of Population Health, University of Oxford OX3 7LF, United Kingdom; f NCDs Prevention and Control Department, Pengzhou CDC, NO.331 Longta Road, Pengzhou, Sichuan, 611930, China; g China National Center for Food Safety Risk Assessment, Beijing 100022, China

**Keywords:** Snoring, Coronary artery disease, Body mass index, Mendelian randomization

## Abstract

**Background and objective:**

Previous observational studies reported a complex relationship between snoring and coronary artery disease (CAD). We aimed to estimate the causal associations between snoring and CAD among East Asians, and the effect independent of BMI.

**Methods:**

Based on 497,250 adults from China Kadoorie Biobank (CKB), we performed a conventional prospective analysis between snoring and CAD outcomes, using the multivariable Cox regression. We also leveraged genome-wide association (GWAS) summary statistics of snoring and BMI from CKB (n=100626, 47,208 snorers) and CAD outcomes from Biobank of Japan (BBJ, 5891~25,892 cases, 142,336~168,186 controls). Single-variable and multivariable two-sample bi-directional Mendelian randomization (MR) analyses were performed.

**Results:**

During a median follow-up of 12.32 years, 48,997 participants developed CAD. Snoring and habitual snoring were associated with incident CAD and myocardial infarction (MI), habitual snoring was also associated with stable angina pectoris (SAP). The HRs (95%CIs) of habitual snoring were 1.06 (1.04, 1.08), 1.06 (1.04, 1.08) and 1.14 (1.03, 1.25). The associations remained among non-adiposity participants. Genetically predicted habitual snoring was associated with CAD and MI, the corresponding IVW-ORs (95%CIs) were 1.09 (1.005, 1.19) and 1.15 (1.05, 1.25). Further adjusted BMI, habitual snoring retained an independent effect on MI and CAD, and showed impacts on SAP (1.09 [1.01,1.17]). No reverse associations were observed between CADs on snoring traits.

**Conclusions:**

Habitual snoring elevated the risks of total CAD, MI, and SAP. The causal associations were independent of BMI. These findings indicated that snoring intervention might contribute to the decrease of CAD risk among Asians.

## Non-standard Abbreviations and Acronyms

CAD: coronary artery disease
MI: myocardial infarction
SAP: stable angina pectoris
CHF: chronic heart failure
UAP: unstable angina pectoris
BMI: body mass index
MR: Mendelian randomization
SVMR: single-variable Mendelian randomization
MVMR: multivariable Mendelian randomization IVW inverse variance weighted
GWAS: Genome-wide association studies
SNP: single nucleotide polymorphism
GI: genetic instruments
CKB: China Kadoorie Biobank
BBJ: Biobank of Japan

## Introduction

Coronary artery disease (CAD) is the leading cause of death worldwide, with approximately 9.1 million deaths in 2019^1^. In China, CAD mortality has risen to 121.6 per one million in urban and 130.1 per one million in rural^2^. Addressing modifiable lifestyle factors can prevent most CADs. Common sleep problems are vital to health in adults, among which snoring could be easily detected due to the bothering noise and treated by weight reduction^3^.

Previous observational studies showed inconsistent relationships between snoring and CAD outcomes. A meta-analysis pooled 13 studies published before 2014 and reported the positive associations between snoring and total CAD (RR = 1.28, 95% confidence interval [CI]: 1.13, 1.45), myocardial infarction (MI) (risk ratio [RR] = 1.40, 95% CI: 1.19,1.65)^4^. However, traditional observation studies could not exclude potential confounders and reverse causation, failing to make causal inferences.

Given that randomized controlled trials (RCT) on snoring exposure might not be ethical or practical, Mendelian randomization (MR) was a proper study design, in which exposure was predicted by genetic instruments (GIs) that were robust to confounders and reverse relationship^5^. A previous MR study between snoring and CAD was conducted among Europeans, they observed that snoring elevated the risk of hypertension and CAD^6^.

Large-scale studies reported a higher prevalence of snoring among Asians (46.9%)^7^ than among Europeans (37.0%)^8^, indicating that addressing snoring problem in the Asian population is essential. Our recently published genome-wide association study (GWAS) showed that the genetic etiology of snoring was different between the East Asian and European ancestries^7^. The genetic-predicted snoring behaviors based on the variants identified among the Europeans might not be appropriate to generate to the East Asian population. Also, the distributions of CAD were different worldwide^9^. Thus, estimating the causal associations of snoring on CAD among East Asians is of great importance.

Body mass index (BMI) is a well-known risk factor for CAD^10^. Meanwhile, higher BMI was a risk factor for snoring, supported by our previous observational^11^ and MR works among Chinese adults^7^. Thus, it’s essential to control the potential confounder, BMI, in the association estimation between snoring and CAD. As BMI shared genetic components with snoring^7,8^, multivariable MR (MVMR) was appropriate to evaluate the causal effects of snoring and BMI independent from each other, by incorporating IVs for both factors in the model^12^.

**Figure 1:**
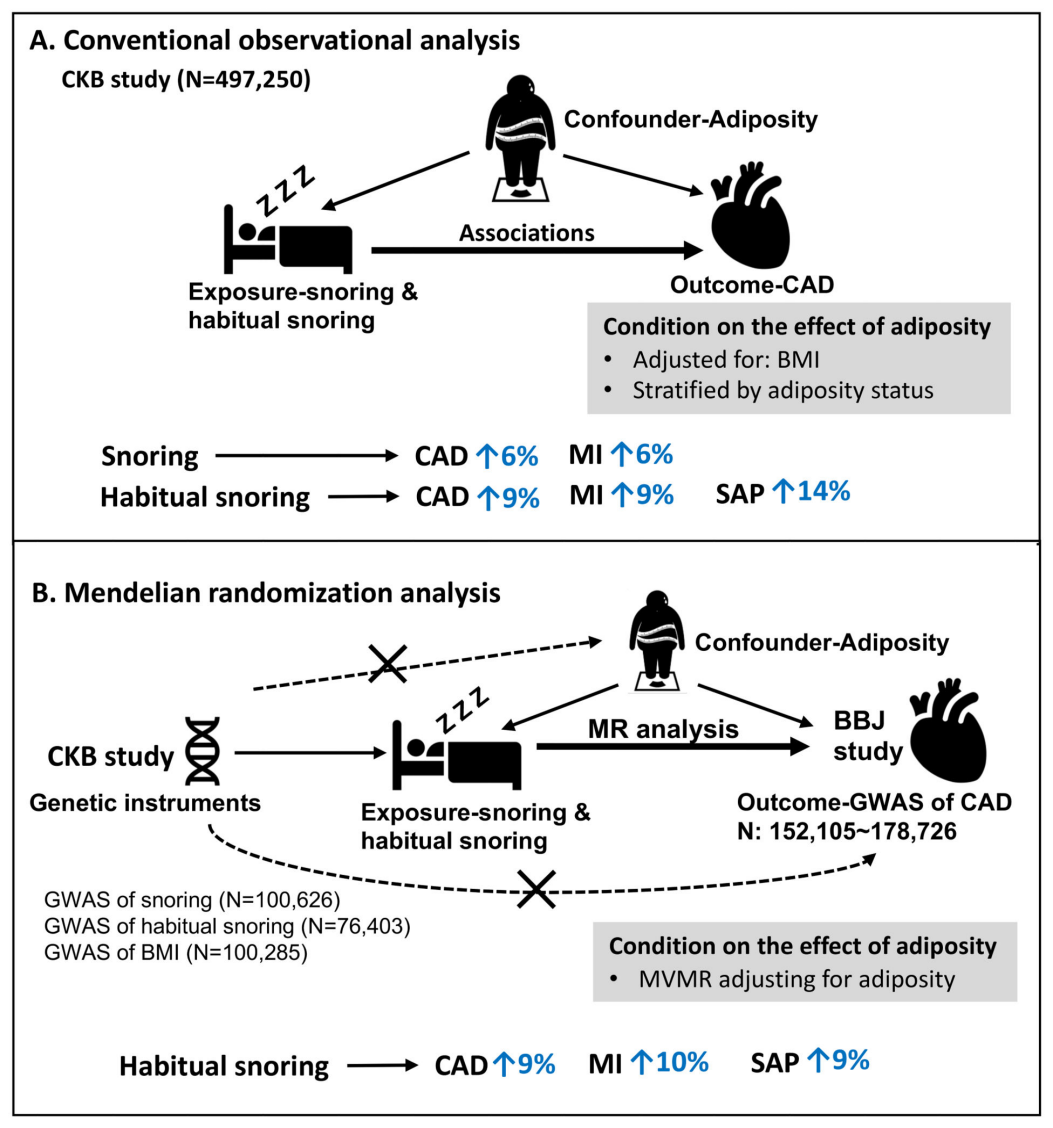
Graphical abstract Notes: CKB, China Kadoorie Biobank; BBJ study; MR, Mendelian randomization; BMI, body mass index; CAD, coronary artery disease; MI, myocardial infarction; SAP, stable angina pectoris. SVMR, single variable MR; MVMR, multivariable MR. The percent in blue meant the elevated risk of CAD per 1.5 of the odds of snoring (for example, an increase in the snoring probability from 20% to 30%). For the conventional observational analysis, we showed the associations in the main analysis, which were robust among the non-adiposity group. For the Mendelian randomization analysis, we showed the associations in the MVMR analysis adjusting for BMI.

We aimed to assess the associations between snoring and CADs, and the effects independent of BMI. Both a conventional prospective analysis among the 0.5 million Chinese adults from the China Kadoorie Biobank (CKB) and the MR analysis leveraging the GWAS summary statistics from CKB and Biobank of Japan (BBJ) was performed (Figure 1).

## Method

### CKB study design and Participants

The CKB cohort recruited 512,725 adults aged 30-79 years living in ten study areas across China, all participants were of East Asian ancestry. Extensive questionnaire data, physical measurements, and blood samples were collected upon baseline assessment in 2004-2008. We excluded those with diagnosed CAD at baseline (N= 15,473) and those with missing BMI (N=2), leaving a total of 497,250 participants for the conventional prospective analysis.

Two batches of participants completed the genotyping procedures (n = 100,640) including 8,143 atherosclerotic vascular diseases, 5,917 hemorrhagic strokes, 5,203 chronic obstructive pulmonary diseases, and 81,377 healthy controls ^13,14^. We excluded participants with mismatches between genetic and reported sex (N=13) or whose BMI data were missing (N=1), leaving 100,626 participants for the genetic analysis.

### Prospective cohort analysis

#### Snoring, BMI, and other characteristics

In the CKB baseline survey, participants were asked about their snoring habits: “Do you snore during sleep?” Three options were available: “Frequently”, “Sometimes”, or “No or Do not know” The present study combined participants reported with the first two options into the snoring group, and others were in the non-snoring group. The CKB study conducted a repeated questionnaire survey within 1-2 weeks after the baseline survey, which involved 15,720 randomly selected participants. The snoring behavior showed good reproducibility (weighted-kappa = 0.69). Weight, height, and blood pressure were measured by trained staff using calibrated instruments^14,15^. BMI was calculated as weight in kilograms divided by height in meters squared.

The baseline survey also collected information of demographic information (age, sex, study regions, highest education, household income, marital status), lifestyle factors (alcohol consumption, smoking, physical activity-metabolic equivalent [MET] h/d), family history of stroke, and heart attack, self-reported diagnoses of diabetes.

### Coronary artery diseases

The CKB study identified incident cases of CAD through linkages to disease and mortality registries as well as the national inpatient health insurance claim database, supplemented with local residential records and annual active confirmation. Six outcomes were included in the present study: MI (I21, I22, I23, I24, I25), SAP (I20.9), UAP (I20.0), CHF (I50.0), angina (I20), CAD (combination of MI, SAP, and UAP). The validity of diagnosed CAD outcomes was described in the Supplementary Methods.

### Descriptive and prospective analysis

#### Genetic instruments for snoring and BMI

A detailed description of genotyping, imputation, and quality control for genetic variants in CKB was shown in the supplemental Methods. Genetic instruments (GIs) for snoring, habitual snoring, and BMI were derived from the GWAS summary statistics in the CKB population based on our previous works^7,15^ (snoring GWAS: 100,626 participants, 47,208 snorers; habitual snoring GWAS: 76,403 participants, 22,985 snorers; BMI GWAS: N=100,285). The present study included SNPs associated at a genome-wide significant level (P < 5×10^-8^) and pruned those with stringent pairwise linkage disequilibrium (LD) (R^2^ > 0.001, window < 10,000kb, based on 1000 genomes project east sample [1000G EAS]). Procedures of GWASs and selection for genetic instruments were shown in the Supplemental Methods.

### CAD GWAS summary statistics

GWAS summary statistics of CAD outcomes and the GIs for CAD were obtained from the BBJ population of East Asian ancestry^16,17^, which were from the publicly available GWAS catalog website (https://www.ebi.ac.uk/gwas/downloads/summary-statistics) and BBJ PheWeb (https://pheweb.jp/). The BBJ participants were independent of CKB. Details of the BBJ study design, genotyping, and quality control were previously described^16–19^. MI (ICD-10 code as I21/I22/I23/I24/I25, 14,992 cases, 146,214 controls), CHF (I50.0, 10,540 cases, 168,186 controls), angina (I20, 14,007 cases, 145,158 controls), UAP (I20.0, 5891 cases, 146,214 controls) and SAP (I20.9, 18,833 cases, 146,214 controls) and CAD (combination of MI, SAP and UAP, 32,512 cases, and 146,214 controls) were included as the outcomes. The criteria for selecting GIs were similar to those above, except that a suggestive significant level (P<1×10^-5^) was applied in the GIs selection for CHF.

### Single variable and multivariable MR

We conducted two-sample bi-directional MR leveraged GWAS summary statistics of snoring, habitual snoring, and BMI from China Kadoorie Biobank (CKB), and GWAS summary statistics of CAD outcomes derived from Biobank Japan (BBJ)^16,17^. The single variable MR (SVMR) was performed to estimate the causal association between snoring traits and CAD outcomes.

As BMI shared genetic components with snoring^7,8^, MVMR was applied to evaluate the causal effects of snoring and BMI independent from each other, by incorporating IVs for both factors in the model. MVMR analysis could estimate the causal effects of snoring and BMI as some specific GIs had stronger effects on a specific exposure than others ^12^. Additionally, MR analysis in the reverse direction was performed to investigate the associations between CADs and snoring traits.

## Results

### Characteristics of CKB participants

Among 497,250 Chinese adults, 48,997 developed CAD, including 48,519 MI cases, 4,388 angina cases, 2,943 SAP cases, 1,153 UAP cases, and 242 CHF cases, during a median follow-up of 12.32 years.

As is shown in Table 1, 46.0% and 21.8% of the CKB participants reported snoring and habitual snoring in the baseline survey. Those with snoring problems are more likely to be males, elders, urban residents, married, with higher household incomes, more likely to drink daily, smoke weekly, with diagnosed diabetes, with family history of stroke and heart attack, more likely to be general adiposity, with higher diastolic blood pressure at baseline (P for trend < 0.001).

### Prospective associations between snoring and CAD

Positive associations were observed between snoring and incident CAD, MI, the corresponding HR (95%CI) in Model 3 were 1.06 (1.04, 1.08) and 1.06 (1.04, 1.08). The associations were robust among the non-adiposity and adiposity groups. Snoring was also associated with higher risks of angina (Model 3: HR=1.07, 95%CI: 1.01, 1.14) and SAP (1.09 [1.01, 1.18]). The associations were diminished to null in the stratified analysis by adiposity status. Additionally, snoring was associated with CHF among the non-adiposity group (Table 2).

Habitual snoring was associated with higher risks of CAD (Model 3: HR [95%CI] = 1.09 [1.07, 1.12]), MI (1.09 [1.07, 1.12]), and SAP (1.14 [1.03, 1.25]). The associations were also observed among the non-adiposity participants, corresponding HRs (95%CIs) in Model 3 were 1.10 (1.07, 1.14), 1.11 (1.07, 1.15), 1.16 (1.01, 1.33). Besides, we observed the association between habitual snoring and angina, both among all and the adiposity participants, and the association with CHF in the non-adiposity group (Table 3).

### Genetic instruments

Three SNPs for snoring, three for habitual snoring, 55 for BMI, 8 to 48 for CADs were selected as the GIs in the SVMR analysis. Besides, 26 and 32 SNPs were respectively selected as proxies for snoring and habitual snoring at suggestively significant level. The *F*-statistics for the individual SNPs for the corresponding exposure were larger than ten, which showed a small magnitude of weak GI bias (Supplementary Table 1-6).

### Impact of snoring, habitual snoring on CAD outcomes

**Figure 2:**
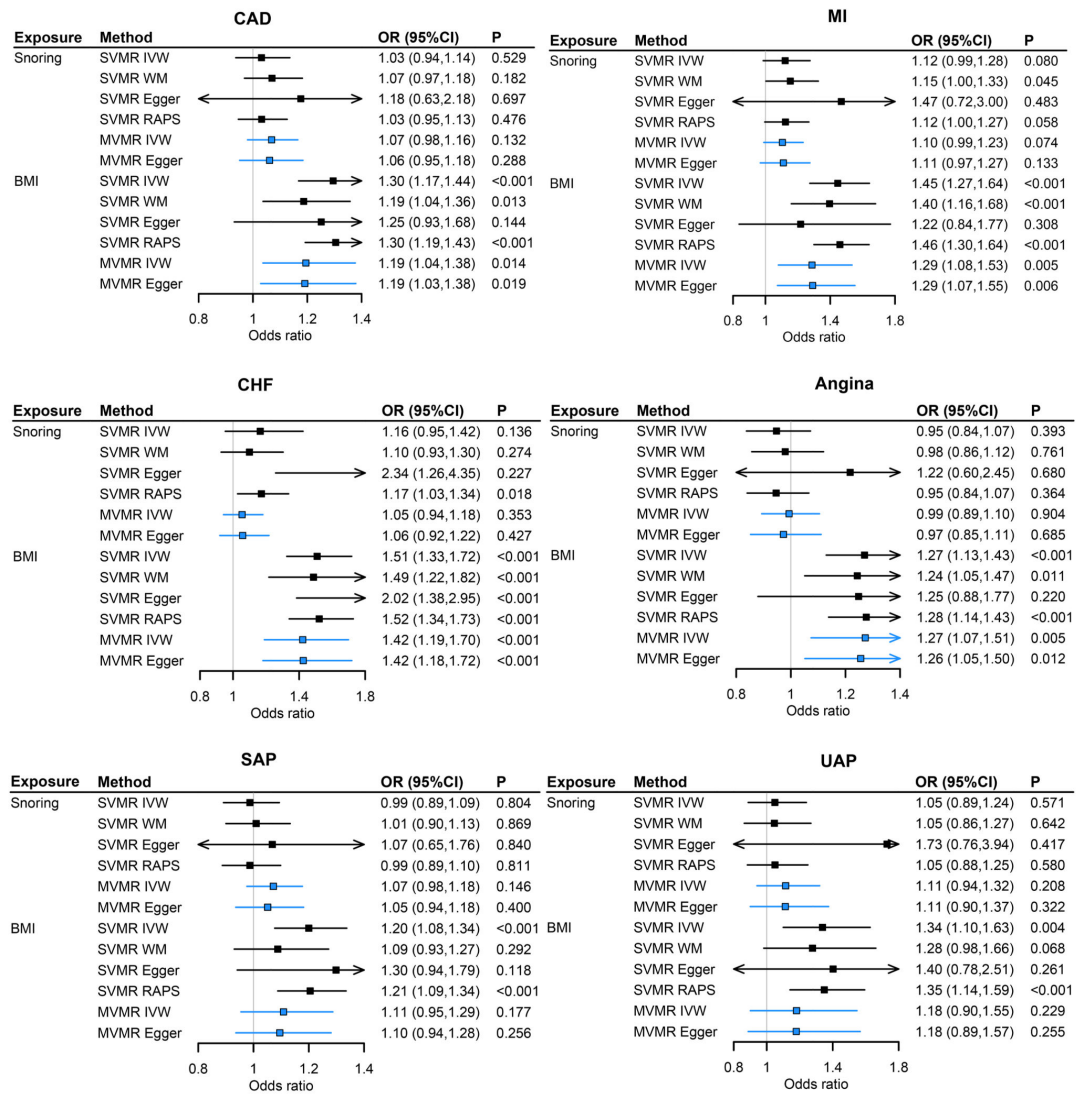
Associations of genetically predicted BMI and snoring with CAD outcomes by SVMR and MVMR. Notes: BMI, body mass index; CAD, coronary artery disease; MI, myocardial infarction; CHF, chronic heart failure; UAP, unstable angina pectoris; SAP, stable angina pectoris; SVMR, single variable MR; MVMR, multivariable MR; IVW, inverse variance weighted; RAPS, MR robust adjusted profile score. For snoring, estimates were expressed per 0.5-fold increase in the probability of snoring (MVMR adjusted for BMI) on the risk of outcomes (CAD, MI, CHF, angina, UAP, SAP). For BMI, estimates were expressed per one SD increase in the BMI (MVMR adjusted for snoring) on the risk of outcomes.

Generally predicted snoring was not associated with risks of CAD outcomes (Figure 2). The results were robust when adjusting for BMI, including GIs for snoring at suggestively significant level (Supplementary Table 7,9).

**Figure 3:**
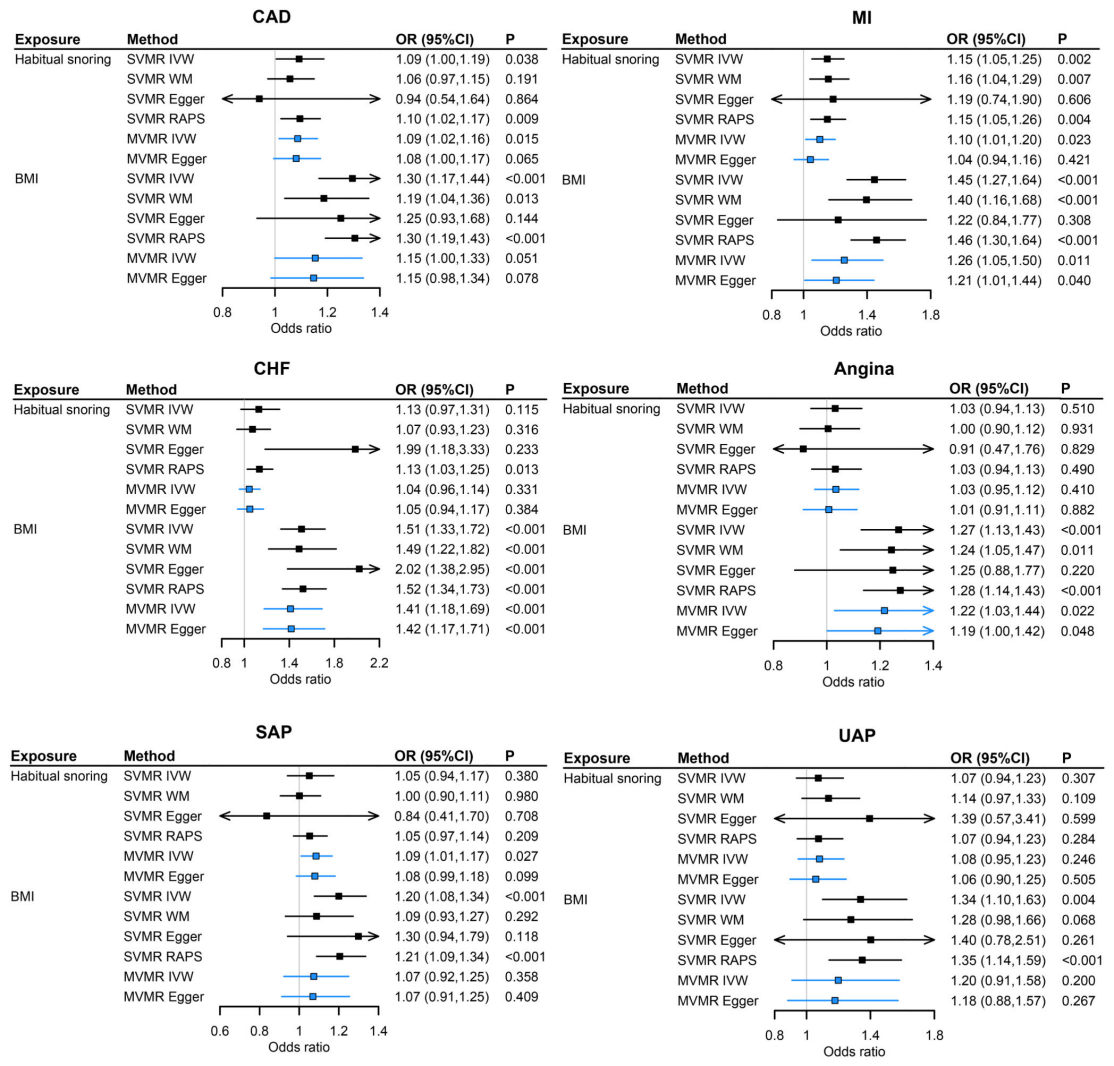
Associations of genetically predicted BMI and habitual snoring with CAD outcomes by SVMR and MVMR. Notes: BMI, body mass index; CAD, coronary artery disease; MI, myocardial infarction; CHF, chronic heart failure; UAP, unstable angina pectoris; SAP, stable angina pectoris; SVMR, single variable MR; MVMR, multivariable MR; IVW, inverse variance weighted; RAPS, MR.robust adjusted profile score. For habitual snoring, estimates were expressed per 0.5-fold increase in the probability of habitual snoring (MVMR adjusted for BMI) on the risk of outcomes (CAD, MI, CHF, angina, UAP, SAP). For BMI, estimates were expressed per one SD increase in the BMI (MVMR adjusted for habitual snoring) on the risk of outcomes.

Genetically predicted per 0.5-fold increased probability of habitual snoring was associated with 9%, and 15% increased risks of CAD and MI, respectively. The corresponding ORs (95%CIs) in IVW analysis were 1.09 (1.005, 1.19) and 1.15 (1.05, 1.25) (Figure 3). Including SNPs associated with habitual snoring at P<1×10^-5^, the sensitivity analysis showed elevated risks of CAD, MI, SAP, and UAP (Supplementary Table 7). Besides, SVMR showed that BMI was associated with higher risks of the six CAD outcomes (Figure 3).

Conditioning on BMI, habitual snoring retained an independent effect on CAD (IVW OR = 1.09, 95% CI: 1.02, 1.16) and MI (1.10 [1.01,1.20]). Additionally, habitual snoring showed an impact on the risk of SAP (1.09 [1.01,1.17]) when adjusting for BMI (Figure 3). The sensitivity analysis including GIs at suggestively significant level showed similar results, except that habitual snoring showed a marginal association with MI (P=0.062), and association with UAP (Supplementary Table 9).

The present SVMR and MVMR did not observe the causal effect of CAD on both snoring traits (Supplementary Table 8,10).

### Tests of MR analysis

For SVMR analysis, Cochran’s *Q* test suggested the possibility of heterogeneity was relatively small (P > 0.05). Random-effect models were applied in analyses that showed significant heterogeneity. All analyses passed the MR-Steiger test (P < 0.05), providing support that the orientation of genetic associations was from the corresponding exposures to outcomes. Besides, the horizontal pleiotropy was not observed (test of MR-Egger intercept P > 0.05) (Supplementary Table 11).

For MVMR analysis, Cochran’s *Q* test suggested heterogeneity within the MR analysis between snoring and UAP, reverse MR analyses, and several sensitivity analyses including GIs at suggestively significant level (P < 0.05), random-effect models were applied for the IVW estimates. There was no significant horizontal pleiotropy (P > 0.05). The variance inflation factors (VIFs) showed no high correlation that influenced the MVMR models (Supplementary Table 12).

## Discussion

Based on 0.5 million participants from China Kadoorie Biobank and large-scale GWAS summary statistics of East Asian ancestry, we performed the conventional prospective cohort study and two-sample MR analysis between snoring and CAD. Both the observational and genetic associations were found between habitual snoring and higher risks of CAD, MI, and SAP, such impacts were robust conditioning on BMI. No reverse causal associations between CADs on BMI or snoring traits were observed.

The traditional cohort study and MR analysis consistently demonstrated that habitual snoring was associated with CAD outcomes. The associations remained in the multivariable Cox model adjusting for BMI, in analysis among non-adiposity participants, and in MVMR analysis accounting for the pleiotropic effect of BMI. These similar effects across the two types of study designs enhanced the reliability of the present results. The observed associations between total snoring and CAD outcomes in the prospective cohort analysis couldn’t be replicated in the MR analysis, probably due to the residual confounding bias in the observational studies, such as the unmeasured metabolic, hormonal, immune biomarkers^20,21^, and the medication history.

The associations between snoring and CAD, MI were in line with a previous meta-analysis including participants from multiple ancestries (N=151,366, RR=1.28, 95%CI: 1.13-1.45)^4^. In addition, the null association between snoring and total angina was in line with the previous observational study^22^. Only one MR study was conducted for the casual estimates between snoring and CAD outcomes among Europeans. Similar to the present study, they did not observe the causal impact of snoring on total CAD (OR=1.30, 95%CI: 0.94, 1.79) or heart failure (1.09 [0.85,1.41]) when adjusting for BMI ^6^.

The present MR study focused on East Asians. We selected three independent SNPs associated with snoring at a genome-wide significant level as the GIs based on our recent GWAS of snoring among the 100,626 CKB participants ^7^. One of the genetic variants applied for snoring was a novel locus (rs712398 mapped on the *SLC25A21* gene) among Chinese adults. Thus, the present GIs could adequately proxy the genetic susceptibility of snoring on risks of CAD outcomes among Asians.

Compared with previous studies, we distinguished differences in the effects of snoring and habitual snoring on CAD. One of the genetic variants applied for habitual snoring (rs140138951 mapped on *BDNF* gene) differed from that for snoring (rs2277339 mapped on *PRIM1* gene) in the present study. The habitual snoring was positively associated with the risk of total CAD, MI, and SAP, while the present study did not observe the effect of snoring. Compared with snoring occasionally, habitual snoring was a severe phenotype that was more likely caused by organic changes in the upper airway^23^, which led to CAD development. Therefore, we should pay more attention to chronic snoring problems to prevent CAD.

Notably, our study extended the subtypes of CAD outcomes compared with the previous MR study. We were the first to investigate the causal effect of snoring on MI and different types of angina, which led to heavy burdens of inpatient occurrences in China ^24,25^. The present result showed that habitual snoring was associated with higher risks of MI and SAP with conditioning on BMI, indicating that intervention in habitual snoring was important for the prevention of CAD hospitalization among Asians.

Considering that some SNPs were associated with both snoring and the body mass index, MVMR was applied to evaluate the causal association for one exposure conditioning on another exposure. The VIFs derived in the present MVMR linear regression models indicated no high collinearity, which ensured the MVMR analysis could evaluate the effects of snoring and BMI, separately^5^. Even if a moderate collinearity existed, it could only inflate the standard errors of the causal estimates, leading the associations toward the null hypothesis^26^.

Causal estimates were slightly different between SVMR and MVMR in the present study. Genetically predicted habitual snoring was associated with increased risks of SAP when adjusting for BMI. The effect was much weaker in the SVMR, suggesting the impact of BMI masked the causal relationship between snoring on total CAD and SAP. More studies are necessary to confirm this hypothesis. As for BMI, SVMR showed the impact of higher BMI on CAD outcomes, which was also in line with previous MR studies^10^. And the effect of BMI was diminished in MVMR analysis. Considering that adiposity was causal for snoring development^8,11^, snoring might act as a mediator in the total effect of BMI on CAD outcomes among the Asian population.

Biological mechanisms underlying the linkage between snoring and CAD outcomes may involve several pathways. Snoring causes negative pressure fluctuation, and unbalance between the supply and demand of oxygen in the left ventricular, contributing to a higher risk of heart disease^4^. Besides, snoring was accompanied by vibration around the carotid artery tissues, leading to damage to endothelial and atherosclerosis^27^. In addition, complete or partial upper airway obstruction was responsible for intermittent hypoxia, oxidative stress, and inflammation response during sleep and was causal for endothelial dysfunction^4^.

To our knowledge, the present study was the first to estimate the causal effect of snoring on CAD outcomes in the Asian population. The conventional observational analysis based on 0.5 million Chinese adults with long-term follow-up and a genetic analysis leveraging GWAS summary statistics of East Asians showed consistent associations between habitual snoring and CAD outcomes, indicating the robustness of our findings. It was also the first time to investigate the effects of genetically predicted probabilities of snoring and habitual snoring traits separately, the loci identified in the Chinese population were used to proxy the snoring traits among Asians. We also applied detailed information on different CAD outcomes, especially focused on MI and angina. In addition, MVMR analysis was conducted to address the genetic correlation between the two exposures^12^. Moreover, the findings highlighted the impact of habitual snoring on CAD, which provided evidence on managing chronic snoring problems.

However, several limitations should be mentioned. First, snoring status was self-reported and might suffer from information bias. Meanwhile, the misclassification tended to be non-differential, leading to the results toward the null hypotheses^28^. Second, GIs selected for snoring traits based on CKB might not comprehensively characterize the causal effect of snoring on CAD development. Therefore, we used strict criteria to select GIs and the *F* statistics were more than ten, suggesting a small magnitude of the weak instrument bias. As the sample size of snoring GWAS in the CKB population is relatively large (N=100,626), we applied the threshold of P < 5 × 10^-8^ in the main analysis, which was recommended to ensure the relevance assumption of MR study^29^. Additionally, the imbalanced number of GIs for snoring versus BMI made it hard to interpret results in MVMR analysis, we applied a relaxed threshold (P <1×10^-5^) for snoring GIs selection in the sensitivity analysis. Most of the findings in the main analysis could be replicated in the sensitivity analysis. Third, there were no available GWAS summary statistics of snoring in BBJ, and we could not replicate the associations of GIs on snoring in the BBJ population. Besides, both the CKB and BBJ participants were of East Asian ancestry, which might limit the discrepancy between the two samples. In addition, considering that applying the corrected significant P value threshold for the multiple CAD outcomes could ignore the possible important causal associations for public health and clinical practice, we applied the conventional P value threshold (P<0.05), the effect sizes, and 95%CIs for the interpretation of our study findings. Our MR estimates do provide a valid test for the possible causal null hypothesis^30^. Nevertheless, more relevant studies are required to strengthen the present causal associations. Last, some key information, such as oxyhemoglobin saturation, metabolic and inflammation markers, was unavailable, so the current study did not further examine the biological pathway of the association between BMI, snoring, and CAD.

## Conclusions

To sum up, the present study found both observational and genetic evidence for a positive impact of habitual snoring on the risks of CAD and MI. Furthermore, accounting for BMI, habitual snoring retained its causal effect and was associated with SAP. Our results suggested that the intervention in habitual snoring problems could be beneficial to the prevention of CAD among East Asians.

## Supplementary Material

Supplementary Tables

## Figures and Tables

**Table 1:** Baseline characteristics of 497,250 participants by snoring status.

Characteristics	No snoring	Occasionally snoring	Habitual snoring
N (%)	268,466 (54.0)	120,474 (24.2)	108,310 (21.8)
Males (%)	91,310 (34.0)	54,696 (45.4)	58,481 (54.0)
Age (years, SD)	50.8 (10.9)	52.1 (10.2)	53.6 (9.9)
Urban (%)	109,183 (40.7)	54,968 (45.6)	51,531 (47.6)
Middle school or above (%)	129,168 (48.1)	64,131 (53.2)	50,944 (47.0)
Household income ≥ 35,000 yuan / year (%)	42,871 (16.0)	24,415 (20.3)	22,264 (20.6)
Married (%)	241,837 (90.1)	110,557 (91.8)	99,099 (91.5)
Current daily smoking (%)	65,391 (24.4)	38,272 (31.8)	43,106 (39.8)
Current weekly drinking (%)	31,098 (11.6)	20,135 (16.7)	23,421 (21.6)
Prevalent diabetes (%)	12,275 (4.6)	6,980 (5.8)	8,346 (7.7)
Family history of stroke (%)	44,059 (16.4)	23,715 (19.7)	21,302 (19.7)
Family history of heart attack (%)	7,855 (2.9)	4,176 (3.5)	3,784 (3.5)
Physical activity in MET (hour/day, SD)	21.5 (13.9)	21.1 (13.8)	21.2 (14.1)
BMI (kg/m^2^, SD)	22.9 (3.1)	23.9 (3.2)	24.9 (3.6)
Non-adiposity (BMI < 24 kg/m^2^, %)	175,257 (65.3)	63,025 (52.3)	44,035 (40.7)
Adiposity (BMI ≥ 24 kg/m^2^, %)	93,209 (34.7)	57,449 (47.7)	64,275 (59.3)
SBP (mmHg, SD)	128.5 (20.9)	131.8 (20.8)	135.5 (21.5)
DBP (mmHg, SD)	76.5 (10.9)	78.3 (11.1)	80.3 (11.4)

For continuous characteristics, plus-minus values are means ± standard deviations (SD). For categorical characteristics, the percentages were shown in the parentheses. Linear (for continuous characteristics) or logistic (for categorical characteristics) models were used to estimate the relationship between each characteristic and snoring status, adjusting for age, sex, and study regions. All the P for trend values <0.001.

**Table 2:** Associations between snoring and incident coronary artery disease.

Coronary artery disease (CAD)		All	Stratified by adiposity (BMI ≥ 24 kg/m^2^)	
			Non-adiposity	Adiposity
CAD				
	Cases	25,183	9,856	15,327
	Incidence rate	9.77	8.16	11.19
	Model 1	1.15 (1.13, 1.17)	1.09 (1.06, 1.12)	1.11 (1.08, 1.14)
	Model 2	1.09 (1.07, 1.11)	1.06 (1.03, 1.08)	1.07 (1.05, 1.10)
	Model 3	1.06 (1.04, 1.08)	1.06 (1.03, 1.09)	1.05 (1.02, 1.08)
MI				
	Cases	24,947	9,783	15,164
	Incidence rate	9.68	8.10	11.07
	Model 1	1.15 (1.13, 1.17)	1.09 (1.06, 1.12)	1.11 (1.08, 1.14)
	Model 2	1.09 (1.08, 1.12)	1.06 (1.03, 1.09)	1.07 (1.05, 1.10)
	Model 3	1.06 (1.04, 1.08)	1.06 (1.03, 1.09)	1.05 (1.02, 1.08)
CHF				
	Cases	133	68	65
	Incidence rate	0.05	0.05	0.05
	Model 1	1.23 (0.95, 1.59)	1.50 (1.05, 2.16)	0.89 (0.61, 1.30)
	Model 2	1.15 (0.89, 1.49)	1.47 (1.02, 2.10)	0.84 (0.58, 1.23)
	Model 3	1.15 (0.88, 1.50)	1.55 (1.07, 2.23)	0.82 (0.56, 1.21)
Angina				
	Cases	2,259	924	1,335
	Incidence rate	0.85	0.75	0.93
	Model 1	1.19 (1.12, 1.26)	1.10 (1.01, 1.19)	1.16 (1.06, 1.27)
	Model 2	1.12 (1.06, 1.19)	1.06 (0.97, 1.15)	1.12 (1.03, 1.23)
	Model 3	1.07 (1.01, 1.14)	1.05 (0.96, 1.15)	1.09 (0.999, 1.20)
UAP				
	Cases	638	203	435
	Incidence rate	0.24	0.16	0.30
	Model 1	1.22 (1.08, 1.37)	1.12 (0.93, 1.35)	1.18 (1.01, 1.38)
	Model 2	1.15 (1.02, 1.30)	1.08 (0.89, 1.30)	1.14 (0.97, 1.33)
	Model 3	1.09 (0.97, 1.23)	1.07 (0.89, 1.30)	1.10 (0.94, 1.29)
SAP				
	Cases	1,513	676	837
	Incidence rate	0.57	0.54	0.58
	Model 1	1.21 (1.13, 1.31)	1.13 (1.02, 1.25)	1.17 (1.04, 1.31)
	Model 2	1.14 (1.05, 1.22)	1.08 (0.98, 1.20)	1.13 (1.005, 1.26)
	Model 3	1.09 (1.01, 1.18)	1.08 (0.98, 1.20)	1.10 (0.99, 1.24)

Notes: Model 1 was stratified by age at baseline (in a 10-year interval), sex, and study regions, and was adjusted for highest education (categorical), household income (categorical), and marital status (categorical) at baseline. Model 2 was further adjusted for alcohol consumption (categorical), smoking (categorical), physical activity (continuous), family history of stroke (categorical), family history of heart attack (categorical), diabetes (categorical), and systolic blood pressure (continuous) at baseline. Model 3 was further adjusted for body mass index (continuous). CAD, coronary artery disease; MI, myocardial infarction; CHF, chronic heart failure; UAP, unstable angina pectoris; SAP, stable angina pectoris. The number of cases in the snoring group is shown in the table. Incidence rate, number of cases / 1000 person-year.

**Table 3:** Associations between habitual snoring and incident coronary artery disease.

Coronary artery disease (CAD)		All			Non-adiposity			Adiposity (BMI ≥ 24 kg/m^2^)	
		Occasional snoring	Habitual snoring		Occasional snoring	Habitual snoring		Occasional snoring	Habitual snoring
CAD									
	Cases	12,649	12,534		5,644	4,212		7005	8322
	Incidence rate	9.28	10.32		7.90	8.54		10.81	11.53
	Model 1	1.08 (1.06, 1.11)	1.23 (1.20, 1.26)		1.05 (1.02, 1.08)	1.14 (1.10, 1.18)		1.05 (1.02, 1.08)	1.17 (1.13, 1.20)
	Model 2	1.05 (1.03, 1.07)	1.15 (1.12, 1.17)		1.03 (0.996, 1.06)	1.10 (1.06, 1.14)		1.03 (1.003, 1.07)	1.11 (1.08, 1.15)
	Model 3	1.03 (1.01, 1.05)	1.09 (1.07, 1.12)		1.03 (0.999, 1.06)	1.10 (1.07, 1.14)		1.02 (0.99, 1.05)	1.07 (1.04, 1.11)
MI									
	Cases	12,530	12,417		5,593	4,190		6937	8227
	Incidence rate	9.19	10.22		7.83	8.49		10.70	11.39
	Model 1	1.08 (1.06, 1.11)	1.23 (1.20, 1.26)		1.05 (1.02, 1.08)	1.15 (1.11, 1.19)		1.05 (1.02, 1.08)	1.17 (1.13, 1.20)
	Model 2	1.05 (1.03, 1.08)	1.15 (1.12, 1.17)		1.03 (0.995, 1.06)	1.10 (1.06, 1.14)		1.03 (1.003, 1.07)	1.11 (1.08, 1.14)
	Model 3	1.03 (1.01, 1.05)	1.09 (1.07, 1.12)		1.03 (0.999, 1.06)	1.11 (1.07, 1.15)		1.02 (0.99, 1.05)	1.07 (1.04, 1.11)
CHF									
	Cases	63	70		32	36		31	34
	Incidence rate	0.04	0.06		0.04	0.07		0.05	0.04
	Model 1	1.19 (0.87, 1.63)	1.27 (0.93, 1.73)		1.35 (0.87, 2.09)	1.69 (1.10, 2.60)		0.94 (0.60, 1.48)	0.85 (0.55, 1.33)
	Model 2	1.14 (0.83, 1.57)	1.16 (0.85, 1.58)		1.33 (0.86, 2.06)	1.63 (1.06, 2.51)		0.92 (0.58, 1.44)	0.78 (0.50, 1.22)
	Model 3	1.14 (0.83, 1.57)	1.15 (0.84, 1.59)		1.39 (0.89, 2.16)	1.73 (1.12, 2.67)		0.90 (0.57, 1.43)	0.76 (0.48, 1.19)
Angina									
	Cases	1,163	1,096		541	383		622	713
	Incidence rate	0.83	0.87		0.74	0.76		0.92	0.95
	Model 1	1.11 (1.04, 1.20)	1.29 (1.19, 1.39)		1.05 (0.95, 1.16)	1.17 (1.04, 1.31)		1.10 (0.99, 1.22)	1.22 (1.10, 1.35)
	Model 2	1.07 (0.998, 1.15)	1.19 (1.10, 1.28)		1.02 (0.92, 1.13)	1.11 (0.99, 1.25)		1.08 (0.97, 1.20)	1.16 (1.05, 1.29)
	Model 3	1.04 (0.97, 1.12)	1.11 (1.03, 1.20)		1.02 (0.92, 1.13)	1.10 (0.98, 1.24)		1.07 (0.96, 1.18)	1.12 (1.01, 1.24)
UAP									
	Cases	313	325		122	81		191	244
	Incidence rate	0.22	0.26		0.17	0.16		0.28	0.32
	Model 1	1.13 (0.98, 1.30)	1.33 (1.15, 1.53)		1.11 (0.89, 1.39)	1.13 (0.88, 1.46)		1.08 (0.89, 1.30)	1.28 (1.07, 1.53)
	Model 2	1.09 (0.95, 1.26)	1.22 (1.05, 1.41)		1.09 (0.87, 1.35)	1.06 (0.82, 1.38)		1.06 (0.88, 1.29)	1.21 (1.01, 1.45)
	Model 3	1.06 (0.92, 1.22)	1.13 (0.98, 1.31)		1.08 (0.87, 1.35)	1.06 (0.82, 1.37)		1.05 (0.86, 1.27)	1.15 (0.96, 1.38)
SAP									
	Cases	803	710		399	277		404	433
	Incidence rate	0.57	0.56		0.54	0.55		0.60	0.57
	Model 1	1.14 (1.04, 1.24)	1.31 (1.20, 1.44)		1.08 (0.96, 1.21)	1.21 (1.06, 1.39)		1.12 (0.98, 1.28)	1.23 (1.07, 1.40)
	Model 2	1.09 (0.998, 1.19)	1.20 (1.09, 1.32)		1.04 (0.92, 1.17)	1.16 (1.01, 1.33)		1.10 (0.96, 1.25)	1.16 (1.01, 1.32)
	Model 3	1.06 (0.97, 1.16)	1.14 (1.03, 1.25)		1.04 (0.92, 1.17)	1.16 (1.01, 1.33)		1.08 (0.95, 1.24)	1.13 (0.98, 1.29)

Notes: Model 1 was stratified by age at baseline (in a 10-year interval), sex, and study regions, and was adjusted for highest education (categorical), household income (categorical), and marital status (categorical) at baseline. Model 2 was further adjusted for alcohol consumption (categorical), smoking (categorical), l activity physical (continuous), family history of stroke (categorical), family history of heart attack (categorical), diabetes (categorical), and systolic blood pressure (continuous) at baseline. Model 3 was further adjusted for body mass index (continuous). CAD, coronary artery disease; MI, myocardial infarction; CHF, chronic heart failure; UAP, unstable angina pectoris; SAP, stable angina pectoris. The number of cases in the snoring group is shown in the table. Incidence rate, number of cases / 1000 person-year.

## Data Availability

The GWAS summary statistics from China Kadoorie Biobank (CKB) in the present study have been deposited in the Genome Variation Map (GVM)^31^ in National Genomics Data Center, Beijing Institute of Genomics, Chinese Academy of Sciences and China National Center for Bioinformation^32^, under the accession number GVP000023. The GWAS summary statistics are publicly available in https://bigd.big.ac.cn/gvm/getProjectDetail?Project=GVP000023. The individual-level data of CKB are controlled-access and are available via an application on request. The access policy and procedures of the CKB data are available at www.ckbiobank.org. GWAS summary statistics of coronary artery diseases outcomes from Biobank of Japan (BBJ) were available from the publicly available GWAS catalog website (https://www.ebi.ac.uk/gwas/downloads/summary-statistics) and BBJ PheWeb (https://pheweb.jp/).

## Summary at a Glance

We performed both a conventional prospective analysis and genetic analysis for the causal estimation of snoring on coronary artery diseases (CADs) among East Asians. Habitual snoring was associated with risks of CAD, myocardial infarction, and stable angina pectoris, independent of body mass index.
