# Supplementary material for: Associations between snoring, body mass index, and coronary artery diseases: Observational and Mendelian randomization study in Asia Snoring, BMI, and CAD among East Asians
Source: Respirology. Author manuscript; Available in PMC 2025 May 6. (PMC7617645; doi:10.1111/resp.14893)
Supplement: Supplementary Tables [file EMS205005-supplement-Supplementary_Tables.docx]

***Contents***

[Supplementary Table S1. Three SNPs for snoring identified in CKB GWAS (n=100,626). 2](#_Toc182949005)

[Supplementary Table S2. Three SNPs for habitual snoring identified in CKB GWAS (n=76,403). 3](#_Toc182949006)

[Supplementary Table S3. 26 SNPs associated with snoring at suggestively significant level (P<1E-05) in CKB GWAS (n=100,626). 4](#_Toc182949007)

[Supplementary Table S4. 32 SNPs associated with habitual snoring at suggestively significant level (P<1E-05) in CKB GWAS (n=76,403). 6](#_Toc182949008)

[Supplementary Table S5. 55 SNPs for BMI identified in CKB GWAS (n= 100,285). 8](#_Toc182949009)

[Supplementary Table S6. SNPs for the CADs identified in the BBJ GWAS used in the reverse MR analysis. 11](#_Toc182949010)

[Supplementary Table S7. SVMR analysis for snoring on CADs applying genetic instruments at suggestively significant level (P<1E-05). 16](#_Toc182949011)

[Supplementary Table S8. Reverse SVMR analysis for CADs on BMI and snoring traits. 18](#_Toc182949012)

[Supplementary Table S9. MVMR analysis for snoring, BMI on CADs using snoring loci at suggestively significant level (P<1E-05). 20](#_Toc182949013)

[Supplementary Table S10. Reverse MVMR analysis for CADs, BMI on the risks of snoring traits. 22](#_Toc182949014)

[Supplementary Table S11. Tests for the single variable MR. 24](#_Toc182949015)

[Supplementary Table S12. Tests for the multivariable MR. 26](#_Toc182949016)

**Table S1. Three SNPs for snoring identified in CKB GWAS (n=100,626).**

| **SNP** | **Chr** | **Position** | **EA** | **OA** | **EAF** | **Nearest gene** | ***β*.Snoring** | **SE.Snoring** | **P.Snoring** | ***F*** | **R^2^** | ***β*.BMI** | **SE.BMI** | **P.BMI** |
| --- | --- | --- | --- | --- | --- | --- | --- | --- | --- | --- | --- | --- | --- | --- |
| rs2277339 | 12 | 57146069 | T | G | 0.760 | *PRIM1* | 0.055 | 0.010 | 4.20E-08 | 29.56 | 0.0001 | 0.020 | 0.005 | 0.00013 |
| rs712398 | 14 | 37385687 | C | T | 0.594 | *SLC25A21* | 0.050 | 0.009 | 1.40E-08 | 32.24 | 0.0001 | -0.010 | 0.005 | 0.027 |
| rs8043757 | 16 | 53813450 | T | A | 0.123 | *FTO* | 0.080 | 0.013 | 1.60E-09 | 35.95 | 0.0001 | 0.101 | 0.007 | 1.60E-50 |

Notes: SNP, single-nucleotide polymorphisms; Chr, chromosome; CKB, China Kadoorie Biobank; GWAS, genome-wide association study; SE, standard error; EA, effect allele; OA, other allele; EAF, effect allele frequency; GWAS was adjusted for age, age^2^, sex, study areas, genotyping array, baseline disease status and PC1-10 in CKB. *F* statistics for each SNP was calculated by (*β*/SE)^1^. We derived R^2^ of each genetic instrument for snoring using the following formula: R^2^=2 * MAF * (1-MAF) * (*β*/SD)^2^. *β* value was derived from the GWAS of snoring, in which we applied the BOLT-LMM software. The effect size of each SNP (*β*) on the quantitative scale, representing the effect of dosage on the genetic liability of snoring, could be converted to the traditional odds ratio using: OR = *β* / (μ * (1 - μ)), where μ = case fraction. SD meant the standard deviation of the effect size, calculated by the standard error of effect size (SE) * $\sqrt{sample size of GWAS of snoring}$. MAF meant the minor allele frequency of the corresponding SNP. Besides, we showed the associations of the snoring SNPs with BMI in CKB GWAS of BMI.

**Table S2. Three SNPs for habitual snoring identified in CKB GWAS (n=76,403).**

| **SNP** | **Chr** | **Position** | **EA** | **OA** | **EAF** | **Nearest gene** | ***β*.Habitual** | **SE.Habitual** | **P.Habitual** | ***F*** | **R^2^** | ***β*.BMI** | **SE.BMI** | **P.BMI** |
| --- | --- | --- | --- | --- | --- | --- | --- | --- | --- | --- | --- | --- | --- | --- |
| rs140138951 | 11 | 27653995 | C | T | 0.557 | *BDNF* | 0.066 | 0.011 | 1.20E-09 | 36.90 | 0.0002 | 0.032 | 0.004 | 8.90E-13 |
| rs62048402 | 16 | 53803223 | G | A | 0.877 | *FTO* | -0.099 | 0.016 | 1.70E-09 | 36.30 | 0.0001 | -0.102 | 0.007 | 1.30E-52 |
| rs712395 | 14 | 37383792 | C | T | 0.595 | *SLC25A21* | 0.067 | 0.011 | 1.60E-09 | 36.45 | 0.0002 | -0.010 | 0.005 | 0.029 |

Notes: SNP, single-nucleotide polymorphisms; Chr, chromosome; CKB, China Kadoorie Biobank; GWAS, genome-wide association study; SE, standard error; EA, effect allele; OA, other allele; EAF, effect allele frequency; GWAS was adjusted for age, age^2^, sex, study areas, genotyping array, baseline disease status and PC1-10 in CKB. *F* statistics for each SNP was calculated by (*β*/SE)^2^. ^1^We derived R^2^ of each genetic instrument for snoring using the following formula: R^2^=2 * MAF * (1-MAF) * (*β*/SD)^2^. *β* value was derived from the GWAS of snoring, in which we applied the BOLT-LMM software. The effect size of each SNP (*β*) on the quantitative scale, representing the effect of dosage on the genetic liability of habitual snoring, could be converted to the traditional odds ratio using: OR = *β* / (μ * (1 - μ)), where μ = case fraction. SD meant the standard deviation of the effect size, calculated by the standard error of effect size (SE) * $\sqrt{sample size of GWAS of snoring}$. MAF meant the minor allele frequency of the corresponding SNP. Besides, we showed the associations of the habitual snoring SNPs with BMI in CKB GWAS of BMI.

**Table S3. 26 SNPs associated with snoring at suggestively significant level (P<1E-05) in CKB GWAS (n=100,626).**

| **SNP** | **Nearest gene** | **EA** | **EAF** | ***β*.snoring** | **SE.snoring** | **P.snoring** | ***F*** | **R^2^** | **BETA.BMI** | **SE.BMI** | **P.BMI** |
| --- | --- | --- | --- | --- | --- | --- | --- | --- | --- | --- | --- |
| rs10886864 | *-* | C | 0.395 | -0.047 | 0.009 | 1.50E-07 | 27.62 | 0.00013 | 0.019 | 0.005 | 2.40E-05 |
| rs117800169 | *LRAT* | T | 0.981 | -0.144 | 0.032 | 5.60E-06 | 20.41 | 0.00001 | -0.005 | 0.016 | 0.7 |
| rs11783086 | *ZHX2* | T | 0.743 | 0.047 | 0.010 | 4.90E-06 | 21.39 | 0.00008 | 0.009 | 0.005 | 0.088 |
| rs12338541 | *IKBKAP* | G | 0.806 | -0.050 | 0.011 | 8.00E-06 | 20.13 | 0.00006 | -0.014 | 0.006 | 0.024 |
| rs140138951 | *BDNF-AS,*  *LINC00678* | C | 0.557 | 0.046 | 0.009 | 1.90E-07 | 27.45 | 0.00013 | 0.032 | 0.004 | 8.90E-13 |
| rs142765916 | *-* | T | 0.981 | -0.161 | 0.036 | 7.40E-06 | 19.96 | 0.00001 | -0.048 | 0.018 | 0.01 |
| rs150702165 | *LOC101927239* | C | 0.989 | -0.245 | 0.053 | 5.70E-06 | 21.25 | 4.62E-06 | -0.051 | 0.027 | 0.06 |
| rs1583544 | *PDHA2* | G | 0.455 | 0.039 | 0.009 | 8.10E-06 | 19.80 | 0.00009 | 0.008 | 0.004 | 0.078 |
| rs1759831 | *TRPM6* | T | 0.843 | -0.057 | 0.012 | 2.70E-06 | 22.25 | 0.00006 | -0.001 | 0.006 | 0.97 |
| rs1928175 | *CASC15* | G | 0.161 | -0.054 | 0.012 | 5.70E-06 | 20.55 | 0.00005 | -0.015 | 0.006 | 0.017 |
| rs2096224 | *TP73* | G | 0.560 | 0.040 | 0.009 | 5.50E-06 | 20.35 | 0.00010 | 0.003 | 0.004 | 0.48 |
| rs2277339 | *PRIM1* | T | 0.760 | 0.055 | 0.010 | 4.20E-08 | 29.56 | 0.00011 | 0.020 | 0.005 | 0.00013 |
| rs34556371 | *SLC25A13* | A | 0.246 | -0.046 | 0.010 | 6.00E-06 | 20.16 | 0.00008 | -0.003 | 0.005 | 0.57 |
| rs3779617 | *GRHL2* | G | 0.902 | -0.076 | 0.015 | 3.20E-07 | 26.63 | 0.00005 | 0.003 | 0.007 | 0.67 |
| rs453539 | *CLDN14* | C | 0.339 | -0.046 | 0.009 | 8.80E-07 | 24.24 | 0.00012 | 0.003 | 0.005 | 0.48 |
| rs4938068 | *HTR3A* | G | 0.166 | 0.055 | 0.012 | 3.70E-06 | 21.65 | 0.00006 | 0.018 | 0.006 | 0.004 |
| rs5992688 | *CECR2* | C | 0.840 | 0.056 | 0.012 | 1.60E-06 | 22.01 | 0.00006 | 0.005 | 0.006 | 0.43 |
| rs62360416 | *CDC20B* | C | 0.730 | 0.047 | 0.010 | 1.80E-06 | 22.94 | 0.00009 | 0.019 | 0.005 | 0.00019 |
| rs7073675 | *VTI1A* | G | 0.730 | 0.044 | 0.010 | 9.90E-06 | 19.78 | 0.00008 | 0.005 | 0.005 | 0.29 |
| rs712398 | *SLC25A21* | C | 0.594 | 0.050 | 0.009 | 1.40E-08 | 32.24 | 0.00015 | -0.010 | 0.005 | 0.027 |
| rs7213130 | *TEX2* | A | 0.975 | -0.134 | 0.028 | 1.50E-06 | 23.48 | 0.00001 | -0.011 | 0.014 | 0.4 |
| rs74882385 | *-* | G | 0.252 | -0.045 | 0.010 | 6.70E-06 | 20.49 | 0.00008 | 0.001 | 0.005 | 0.87 |
| rs7605371 | *SATB2* | G | 0.642 | -0.043 | 0.009 | 2.90E-06 | 22.70 | 0.00010 | 0.002 | 0.005 | 0.49 |
| rs8043757 | *FTO* | A | 0.877 | -0.080 | 0.013 | 1.60E-09 | 35.95 | 0.00008 | -0.101 | 0.007 | 1.60E-50 |
| rs9643508 | *-* | T | 0.671 | -0.046 | 0.009 | 8.40E-07 | 24.22 | 0.00011 | -0.013 | 0.005 | 0.0081 |
| rs9910905 | *CACNG4* | C | 0.875 | 0.063 | 0.014 | 4.20E-06 | 21.74 | 0.00004 | 0.013 | 0.007 | 0.071 |

Notes: SNP, single-nucleotide polymorphisms; Chr, chromosome; CKB, China Kadoorie Biobank; GWAS, genome-wide association study; SE, standard error; EA, effect allele; OA, other allele; EAF, effect allele frequency; GWAS was adjusted for age, age^2^, sex, study areas, genotyping array, baseline disease status and PC1-10 in CKB. *F* statistics for each SNP was calculated by (*β*/SE)^2^. ^1^We derived R^2^ of each genetic instrument for snoring using the following formula: R^2^=2 * MAF * (1-MAF) * (*β*/SD)^2^. *β* value was derived from the GWAS of snoring, in which we applied the BOLT-LMM software. The effect size of each SNP (*β*) on the quantitative scale, representing the effect of dosage on the genetic liability of snoring, could be converted to the traditional odds ratio using: OR = *β* / (μ * (1 - μ)), where μ = case fraction. SD meant the standard deviation of the effect size, calculated by the standard error of effect size (SE) * $\sqrt{sample size of GWAS of snoring}$. MAF meant the minor allele frequency of the corresponding SNP. Besides, we showed the associations of the snoring SNPs with BMI in CKB GWAS of BMI.

**Table S4. 32 SNPs associated with habitual snoring at suggestively significant level (P<1E-05) in CKB GWAS (n=76,403).**

| **SNP** | **Nearest gene** | **EA** | **EAF** | ***β*.habitual** | **SE.habitual** | **P.habitual** | ***F*** | **R^2^** | **BETA.BMI** | **SE.BMI** | **P.BMI** |
| --- | --- | --- | --- | --- | --- | --- | --- | --- | --- | --- | --- |
| rs10185460 | *NPPC* | T | 0.573 | 0.049 | 0.011 | 6.60E-06 | 20.32 | 0.00013 | 0.023 | 0.004 | 1.70E-07 |
| rs10740058 | *ARID5B* | T | 0.270 | 0.055 | 0.012 | 7.50E-06 | 20.07 | 0.00011 | 0.008 | 0.005 | 0.099 |
| rs10757875 | *-* | C | 0.463 | -0.050 | 0.011 | 3.60E-06 | 21.48 | 0.00013 | -0.005 | 0.004 | 0.25 |
| rs11196089 | *VTI1A* | T | 0.717 | 0.056 | 0.012 | 3.00E-06 | 21.80 | 0.00012 | 0.000 | 0.005 | 0.88 |
| rs1159025 | *-* | A | 0.252 | -0.056 | 0.012 | 8.60E-06 | 19.80 | 0.00011 | 0.003 | 0.005 | 0.58 |
| rs137985456 | *ZNF71* | C | 0.985 | 0.257 | 0.052 | 9.30E-07 | 24.08 | 0.00001 | 0.039 | 0.022 | 0.068 |
| rs140138951 | *BDNF-AS, LINC00678* | C | 0.557 | 0.066 | 0.011 | 1.20E-09 | 36.90 | 0.00023 | 0.032 | 0.004 | 8.90E-13 |
| rs142765916 | *-* | T | 0.981 | -0.201 | 0.045 | 7.40E-06 | 20.10 | 0.00001 | -0.048 | 0.018 | 0.01 |
| rs146583707 | *IL21* | T | 0.883 | -0.080 | 0.017 | 2.20E-06 | 22.43 | 0.00006 | -0.012 | 0.007 | 0.066 |
| rs149392885 | *-* | G | 0.974 | -0.179 | 0.037 | 1.00E-06 | 23.86 | 0.00002 | -0.044 | 0.015 | 0.0036 |
| rs150369882 | *HIVEP3* | G | 0.980 | -0.186 | 0.042 | 8.90E-06 | 19.75 | 0.00001 | 0.018 | 0.017 | 0.31 |
| rs1759831 | *TRPM6* | T | 0.844 | -0.066 | 0.015 | 9.40E-06 | 19.64 | 0.00007 | -0.001 | 0.006 | 0.97 |
| rs1928175 | *CASC15* | G | 0.161 | -0.066 | 0.015 | 8.30E-06 | 19.87 | 0.00007 | -0.015 | 0.006 | 0.017 |
| rs211830 | *-* | G | 0.592 | -0.055 | 0.011 | 6.20E-07 | 24.87 | 0.00016 | -0.007 | 0.005 | 0.082 |
| rs2277339 | *PRIM1* | T | 0.759 | 0.059 | 0.013 | 2.30E-06 | 22.36 | 0.00010 | 0.020 | 0.005 | 0.00013 |
| rs2411195 | *ZNHIT3* | G | 0.679 | 0.052 | 0.012 | 7.30E-06 | 20.13 | 0.00011 | 0.015 | 0.005 | 0.0019 |
| rs25879 | *CSF2* | A | 0.412 | 0.053 | 0.011 | 1.20E-06 | 23.55 | 0.00015 | 0.007 | 0.004 | 0.087 |
| rs2608528 | *ZNF578* | G | 0.703 | 0.060 | 0.012 | 2.90E-07 | 26.32 | 0.00014 | 0.009 | 0.005 | 0.045 |
| rs3264 | *MPDZ* | T | 0.844 | 0.072 | 0.015 | 1.90E-06 | 22.74 | 0.00008 | 0.002 | 0.006 | 0.84 |
| rs376218 | *CLDN14* | A | 0.350 | -0.050 | 0.011 | 9.10E-06 | 19.70 | 0.00012 | 0.004 | 0.005 | 0.35 |
| rs4659140 | *WARS2* | T | 0.487 | 0.049 | 0.011 | 4.90E-06 | 20.88 | 0.00013 | 0.006 | 0.004 | 0.15 |
| rs6132477 | *LOC100270679* | C | 0.359 | 0.052 | 0.011 | 4.80E-06 | 20.91 | 0.00013 | 0.001 | 0.005 | 0.91 |
| rs62048402 | *FTO* | G | 0.877 | -0.099 | 0.016 | 1.70E-09 | 36.30 | 0.00011 | -0.102 | 0.007 | 1.30E-52 |
| rs62446486 | *TNS3* | C | 0.695 | 0.057 | 0.012 | 1.30E-06 | 23.37 | 0.00013 | 0.005 | 0.005 | 0.33 |
| rs6876815 | *ABLIM3* | C | 0.508 | 0.048 | 0.011 | 8.70E-06 | 19.78 | 0.00012 | 0.007 | 0.004 | 0.11 |
| rs712395 | *SLC25A21* | C | 0.595 | 0.067 | 0.011 | 1.60E-09 | 36.45 | 0.00023 | -0.010 | 0.005 | 0.029 |
| rs7195443 | *KIAA0513* | A | 0.414 | 0.051 | 0.011 | 3.90E-06 | 21.32 | 0.00014 | 0.003 | 0.005 | 0.48 |
| rs73003756 | *-* | T | 0.970 | -0.164 | 0.032 | 3.30E-07 | 26.09 | 0.00002 | -0.053 | 0.013 | 6.50E-05 |
| rs754227 | *-* | T | 0.654 | 0.057 | 0.011 | 6.20E-07 | 24.86 | 0.00016 | -0.014 | 0.005 | 0.0021 |
| rs7979584 | *LOC100507065, MSRB3* | T | 0.495 | -0.053 | 0.011 | 7.90E-07 | 24.39 | 0.00015 | 0.011 | 0.004 | 0.011 |
| rs8093341 | *-* | C | 0.201 | -0.066 | 0.014 | 1.40E-06 | 23.27 | 0.00009 | -0.006 | 0.006 | 0.3 |
| rs982931 | *TNR* | C | 0.975 | 0.172 | 0.036 | 1.80E-06 | 22.75 | 0.00001 | 0.022 | 0.015 | 0.16 |

Notes: SNP, single-nucleotide polymorphisms; Chr, chromosome; CKB, China Kadoorie Biobank; GWAS, genome-wide association study; SE, standard error; EA, effect allele; OA, other allele; EAF, effect allele frequency; GWAS was adjusted for age, age^2^, sex, study areas, genotyping array, baseline disease status and PC1-10 in CKB. *F* statistics for each SNP was calculated by (*β*/SE)^2^. We derived R^2^ of each genetic instrument for snoring using the following formula: R^2^=2 * MAF * (1-MAF) * (*β*/SD)^2^. *β* value was derived from the GWAS of snoring, in which we applied the BOLT-LMM software. The effect size of each SNP (*β*) on the quantitative scale, representing the effect of dosage on the genetic liability of habitual snoring, could be converted to the traditional odds ratio using: OR = *β* / (μ * (1 - μ)), where μ = case fraction. SD meant the standard deviation of the effect size, calculated by the standard error of effect size (SE) * $\sqrt{sample size of GWAS of snoring}$. MAF meant the minor allele frequency of the corresponding SNP. Besides, we showed the associations of the habitual snoring SNPs with BMI in CKB GWAS of BMI^1^.

**Table S5. 55 SNPs for BMI identified in CKB GWAS (n= 100,285).**

| **SNP** | **Chr** | **Position** | **EA** | **OA** | **EAF** | ***β*.BMI** | **SE.BMI** | **P.BMI** | ***F*** | **R^2^** | ***β*.Snoring** | **P.Snoring** | ***β*.Habitual** | **P.Habitual** |
| --- | --- | --- | --- | --- | --- | --- | --- | --- | --- | --- | --- | --- | --- | --- |
| rs10080749 | 6 | 34218165 | A | G | 0.890 | -0.049 | 0.007 | 3.20E-12 | 48.04 | 0.0001 | -0.012 | 0.44 | -0.011 | 0.54 |
| rs10133709 | 14 | 103310758 | A | T | 0.611 | -0.032 | 0.005 | 7.10E-13 | 49.81 | 0.0002 | -0.017 | 0.061 | -0.027 | **0.014** |
| rs1055996 | 14 | 92527399 | A | G | 0.688 | -0.032 | 0.005 | 2.80E-11 | 44.96 | 0.0002 | 0.006 | 0.48 | 0.017 | 0.14 |
| rs11039324 | 11 | 47665686 | G | A | 0.687 | -0.026 | 0.005 | 3.60E-08 | 29.86 | 0.0001 | -0.011 | 0.24 | -0.011 | 0.35 |
| rs11248548 | 10 | 125243861 | C | T | 0.703 | 0.035 | 0.005 | 5.90E-14 | 53.53 | 0.0002 | -0.022 | **0.024** | -0.025 | **0.033** |
| rs11642015 | 16 | 53802494 | C | T | 0.875 | -0.103 | 0.007 | 2.30E-53 | 235.13 | 0.0005 | -0.078 | **3.70E-09** | -0.098 | **2.30E-09** |
| rs12433202 | 14 | 23822683 | G | A | 0.363 | 0.027 | 0.005 | 5.00E-09 | 34.51 | 0.0001 | 0.011 | 0.23 | 0.005 | 0.65 |
| rs12587992 | 14 | 36146145 | T | C | 0.923 | 0.061 | 0.009 | 2.00E-13 | 51.68 | 0.0001 | 0.026 | 0.12 | 0.040 | 0.053 |
| rs12618252 | 2 | 25207950 | G | A | 0.464 | 0.039 | 0.004 | 3.70E-19 | 79.23 | 0.0005 | 0.005 | 0.54 | -0.003 | 0.78 |
| rs12719311 | 5 | 122755334 | G | A | 0.579 | -0.025 | 0.005 | 2.80E-08 | 29.80 | 0.0001 | -0.022 | **0.012** | -0.046 | **2.70E-05** |
| rs13047416 | 21 | 40309436 | C | G | 0.422 | 0.028 | 0.005 | 1.10E-09 | 37.43 | 0.0002 | 0.012 | 0.17 | 0.021 | 0.059 |
| rs13130484 | 4 | 45175691 | C | T | 0.700 | -0.040 | 0.005 | 8.50E-17 | 68.82 | 0.0003 | -0.012 | 0.19 | -0.018 | 0.12 |
| rs1361511 | 20 | 17271705 | C | G | 0.474 | -0.026 | 0.004 | 7.80E-09 | 33.48 | 0.0002 | -0.016 | 0.065 | -0.013 | 0.23 |
| rs1514174 | 1 | 74993063 | C | T | 0.842 | 0.040 | 0.006 | 1.80E-11 | 44.55 | 0.0001 | 0.021 | 0.081 | 0.025 | 0.087 |
| rs1532127 | 19 | 47571938 | G | A | 0.706 | -0.031 | 0.005 | 1.30E-10 | 40.36 | 0.0002 | -0.017 | 0.064 | -0.025 | **0.034** |
| rs1561042 | 15 | 47907160 | G | A | 0.488 | 0.031 | 0.004 | 1.10E-11 | 47.62 | 0.0003 | 0.026 | **0.0037** | 0.031 | **0.0042** |
| rs1927861 | 13 | 54091759 | C | T | 0.750 | -0.036 | 0.005 | 6.10E-12 | 48.35 | 0.0002 | -0.018 | 0.08 | -0.030 | **0.016** |
| rs2023885 | 17 | 46126373 | G | A | 0.761 | -0.038 | 0.005 | 4.90E-13 | 52.57 | 0.0002 | 0.002 | 0.88 | 0.000 | 0.99 |
| rs2049008 | 9 | 97236788 | A | G | 0.569 | 0.029 | 0.004 | 9.40E-11 | 41.16 | 0.0003 | 0.008 | 0.38 | 0.010 | 0.35 |
| rs2102808 | 2 | 169117025 | G | T | 0.668 | -0.030 | 0.005 | 2.80E-10 | 39.82 | 0.0002 | -0.022 | **0.018** | -0.023 | **0.04** |
| rs2206271 | 6 | 50786008 | T | A | 0.655 | -0.036 | 0.005 | 5.10E-15 | 59.96 | 0.0002 | -0.025 | **0.0075** | -0.034 | **0.0029** |
| rs2238435 | 16 | 4014282 | C | G | 0.665 | -0.034 | 0.005 | 1.50E-12 | 51.20 | 0.0002 | -0.014 | 0.15 | -0.019 | 0.091 |
| rs2238689 | 19 | 46178661 | T | C | 0.381 | 0.028 | 0.005 | 3.80E-10 | 38.29 | 0.0001 | 0.018 | **0.041** | 0.026 | **0.019** |
| rs2241423 | 15 | 68086838 | G | A | 0.398 | 0.036 | 0.005 | 2.10E-15 | 62.90 | 0.0002 | 0.002 | 0.81 | 0.003 | 0.76 |
| rs2510032 | 11 | 77968631 | G | C | 0.606 | 0.032 | 0.005 | 1.80E-12 | 49.53 | 0.0002 | 0.043 | **1.90E-06** | 0.046 | **2.90E-05** |
| rs31127 | 5 | 53314757 | A | G | 0.162 | 0.037 | 0.006 | 1.00E-09 | 38.32 | 0.0001 | 0.021 | 0.071 | 0.016 | 0.28 |
| rs34719836 | 12 | 122217559 | C | T | 0.975 | 0.085 | 0.014 | 7.40E-10 | 36.75 | 1.79E-05 | 0.004 | 0.9 | -0.001 | 0.98 |
| rs3757894 | 8 | 27996440 | G | C | 0.663 | 0.028 | 0.005 | 8.10E-09 | 34.09 | 0.0001 | 0.013 | 0.16 | 0.020 | 0.085 |
| rs3784635 | 15 | 62254989 | T | C | 0.786 | 0.034 | 0.005 | 4.50E-10 | 39.41 | 0.0002 | 0.017 | 0.098 | 0.020 | 0.12 |
| rs3807648 | 7 | 77828434 | T | C | 0.435 | -0.029 | 0.004 | 4.60E-11 | 42.56 | 0.0003 | 0.009 | 0.3 | 0.000 | 0.97 |
| rs3814424 | 5 | 87968953 | C | T | 0.502 | -0.027 | 0.004 | 5.30E-10 | 37.58 | 0.0002 | -0.010 | 0.22 | -0.009 | 0.4 |
| rs4409766 | 10 | 104616663 | T | C | 0.710 | -0.037 | 0.005 | 3.70E-14 | 57.41 | 0.0002 | -0.007 | 0.47 | -0.004 | 0.73 |
| rs4420537 | 16 | 20257039 | T | C | 0.269 | -0.037 | 0.005 | 8.70E-14 | 53.57 | 0.0002 | -0.019 | 0.06 | -0.027 | **0.025** |
| rs4712523 | 6 | 20657564 | A | G | 0.583 | 0.031 | 0.004 | 1.10E-11 | 46.22 | 0.0003 | 0.007 | 0.46 | 0.008 | 0.45 |
| rs4735692 | 8 | 76615663 | A | G | 0.386 | 0.032 | 0.005 | 2.70E-12 | 48.24 | 0.0002 | 0.019 | **0.034** | 0.031 | **0.0051** |
| rs4973506 | 2 | 232943097 | C | T | 0.488 | -0.027 | 0.004 | 1.80E-09 | 36.70 | 0.0002 | -0.024 | **0.006** | -0.034 | **0.0018** |
| rs571567 | 1 | 177872905 | G | A | 0.815 | -0.061 | 0.006 | 2.90E-27 | 114.15 | 0.0003 | -0.045 | **6.30E-05** | -0.049 | **0.00049** |
| rs59153459 | 1 | 151401045 | G | A | 0.594 | -0.029 | 0.005 | 2.60E-10 | 39.89 | 0.0002 | 0.001 | 0.89 | -0.014 | 0.19 |
| rs61828917 | 1 | 173580303 | C | T | 0.731 | -0.028 | 0.005 | 4.20E-08 | 29.78 | 0.0001 | -0.035 | **0.00039** | -0.036 | **0.0036** |
| rs6439340 | 3 | 131797500 | A | C | 0.843 | -0.036 | 0.006 | 3.70E-09 | 34.50 | 0.0001 | -0.002 | 0.87 | -0.016 | 0.28 |
| rs6731872 | 2 | 624205 | T | G | 0.087 | -0.088 | 0.008 | 5.20E-29 | 126.25 | 0.0002 | -0.032 | **0.039** | -0.067 | **0.00045** |
| rs6882366 | 5 | 95864693 | C | T | 0.423 | 0.032 | 0.005 | 1.20E-12 | 50.14 | 0.0002 | 0.019 | **0.031** | 0.032 | **0.0033** |
| rs7132908 | 12 | 50263148 | G | A | 0.776 | -0.038 | 0.005 | 2.30E-13 | 52.27 | 0.0002 | -0.021 | **0.036** | -0.021 | 0.097 |
| rs72639513 | 10 | 31786358 | G | A | 0.845 | 0.035 | 0.006 | 8.60E-09 | 32.08 | 0.0001 | 0.039 | **0.0013** | 0.043 | **0.0042** |
| rs73168038 | 3 | 161409329 | C | T | 0.808 | -0.036 | 0.006 | 4.30E-10 | 39.39 | 0.0001 | -0.016 | 0.14 | -0.037 | **0.0073** |
| rs75868869 | 17 | 66098154 | A | G | 0.613 | 0.028 | 0.005 | 6.60E-10 | 37.80 | 0.0001 | -0.003 | 0.71 | 0.002 | 0.85 |
| rs76152047 | 3 | 141183792 | A | G | 0.759 | -0.029 | 0.005 | 3.00E-08 | 31.13 | 0.0001 | 0.005 | 0.62 | 0.017 | 0.18 |
| rs762057 | 22 | 33950513 | G | A | 0.732 | -0.030 | 0.005 | 2.70E-09 | 36.51 | 0.0001 | -0.020 | **0.049** | -0.024 | 0.054 |
| rs79553048 | 2 | 58920057 | G | A | 0.850 | -0.044 | 0.006 | 1.20E-12 | 48.28 | 0.0001 | -0.012 | 0.3 | -0.010 | 0.51 |
| rs8049326 | 16 | 3572268 | G | A | 0.610 | -0.028 | 0.005 | 2.30E-09 | 36.56 | 0.0001 | -0.012 | 0.16 | -0.015 | 0.17 |
| rs9312002 | 3 | 8137447 | G | A | 0.620 | -0.028 | 0.005 | 1.20E-09 | 38.46 | 0.0001 | -0.009 | 0.29 | -0.009 | 0.41 |
| rs9528957 | 13 | 66243748 | G | A | 0.230 | -0.029 | 0.005 | 3.00E-08 | 30.75 | 0.0001 | -0.021 | **0.046** | -0.024 | 0.059 |
| rs979873 | 3 | 42356679 | A | G | 0.823 | 0.043 | 0.006 | 2.70E-14 | 55.93 | 0.0001 | 0.011 | 0.34 | 0.019 | 0.17 |
| rs9815805 | 3 | 115102814 | G | A | 0.366 | -0.026 | 0.005 | 1.10E-08 | 32.41 | 0.0001 | -0.001 | 0.9 | -0.003 | 0.8 |
| rs9816226 | 3 | 185834499 | A | T | 0.046 | -0.066 | 0.011 | 5.20E-10 | 38.26 | 3.15E-05 | -0.034 | 0.12 | -0.056 | **0.03** |

Notes: SNP, single-nucleotide polymorphisms; Chr, chromosome; CKB, China Kadoorie Biobank; GWAS, genome-wide association study; SE, standard error; EA, effect allele; OA, other allele; EAF, effect allele frequency; GWAS was adjusted for genotyping array and PC1-10 in CKB, using the inverse normal transformed residual of BMI adjusting for age, age^2^, sex, study areas. *F* statistics for each SNP was calculated by (*β*/SE)^2^. We derived R^2^ of each genetic instrument for snoring using the following formula: R^2^=2 * MAF * (1-MAF) * (*β*/SD)^2^. *β* value was derived from the GWAS of snoring, in which we applied the BOLT-LMM software. SD meant the standard deviation of the effect size, calculated by the standard error of effect size (SE) * $\sqrt{sample size of GWAS of snoring}$. MAF meant the minor allele frequency of the corresponding SNP. Besides, we showed the associations of the BMI SNPs with snoring and habitual snoring in the corresponding CKB GWAS. rs476828, rs6265 were excluded for they were previously reported to be associated with coronary heart disease.

**Table S6. SNPs for the CADs identified in the BBJ GWAS used in the reverse MR analysis.**

| **SNP** | **EA** | **OA** | ***β*** | **SE** | ***F*** | **R^2^** | **P** | **EAF** | **N** |
| --- | --- | --- | --- | --- | --- | --- | --- | --- | --- |
| **Angina** |  | | | | | | | | |
| rs10757274 | G | A | 0.155 | 0.013 | 135.83 | 0.00045 | 2.17E-31 | 0.472 | 159165 |
| rs11107904 | C | T | -0.089 | 0.016 | 32.08 | 0.00007 | 1.48E-08 | 0.231 | 159165 |
| rs11227229 | A | G | 0.080 | 0.013 | 35.97 | 0.00012 | 2.00E-09 | 0.434 | 159165 |
| rs115740773 | A | G | 0.174 | 0.027 | 40.45 | 0.00003 | 2.02E-10 | 0.066 | 159165 |
| rs116873087 | C | G | 0.152 | 0.017 | 77.51 | 0.00018 | 1.32E-18 | 0.238 | 159165 |
| rs1384705 | T | C | -0.088 | 0.014 | 42.12 | 0.00012 | 8.60E-11 | 0.389 | 159165 |
| rs17115100 | T | G | -0.083 | 0.014 | 35.10 | 0.00010 | 3.13E-09 | 0.336 | 159165 |
| rs1909196 | C | T | 0.085 | 0.013 | 39.59 | 0.00013 | 3.14E-10 | 0.452 | 159165 |
| rs2327429 | C | T | -0.098 | 0.013 | 55.07 | 0.00018 | 1.17E-13 | 0.520 | 159165 |
| rs2738464 | C | G | 0.080 | 0.014 | 31.58 | 0.00009 | 1.92E-08 | 0.685 | 159165 |
| rs4932463 | G | T | -0.091 | 0.013 | 46.50 | 0.00015 | 9.16E-12 | 0.546 | 159165 |
| rs6841473 | T | C | 0.085 | 0.014 | 35.08 | 0.00010 | 3.16E-09 | 0.312 | 159165 |
| rs75346744 | G | A | 0.168 | 0.028 | 36.67 | 0.00003 | 1.40E-09 | 0.061 | 159165 |
| rs79717953 | T | C | 0.138 | 0.025 | 30.89 | 0.00003 | 2.73E-08 | 0.083 | 159165 |
| rs9349379 | G | A | 0.100 | 0.015 | 42.93 | 0.00013 | 5.68E-11 | 0.649 | 159165 |
| **CAD** |  | | | | | | | | |
| rs10029651 | T | C | -0.057 | 0.010 | 29.81 | 0.00008 | 4.78E-08 | 0.328 | 178726 |
| rs1054111 | A | C | -0.072 | 0.010 | 52.84 | 0.00014 | 3.63E-13 | 0.414 | 178726 |
| rs10757274 | G | A | 0.167 | 0.010 | 292.44 | 0.00078 | 1.46E-65 | 0.476 | 178726 |
| rs11105377 | T | C | -0.067 | 0.010 | 43.90 | 0.00012 | 3.45E-11 | 0.625 | 178726 |
| rs11107908 | T | C | -0.091 | 0.012 | 61.07 | 0.00011 | 5.52E-15 | 0.230 | 178726 |
| rs11191447 | T | C | -0.097 | 0.012 | 70.92 | 0.00014 | 3.72E-17 | 0.246 | 178726 |
| rs1144789 | G | A | -0.062 | 0.010 | 38.55 | 0.00010 | 5.32E-10 | 0.613 | 178726 |
| rs116873087 | C | G | 0.221 | 0.013 | 309.04 | 0.00059 | 3.54E-69 | 0.243 | 178726 |
| rs117123860 | C | T | 0.127 | 0.023 | 29.86 | 0.00002 | 4.65E-08 | 0.057 | 178726 |
| rs117598591 | A | C | 0.191 | 0.019 | 101.01 | 0.00007 | 9.15E-24 | 0.071 | 178726 |
| rs11925504 | A | G | -0.077 | 0.012 | 38.57 | 0.00008 | 5.28E-10 | 0.211 | 178726 |
| rs12225799 | G | C | 0.090 | 0.015 | 37.42 | 0.00004 | 9.52E-10 | 0.125 | 178726 |
| rs13281248 | A | G | -0.099 | 0.018 | 31.03 | 0.00003 | 2.54E-08 | 0.098 | 178726 |
| rs1412445 | T | C | 0.072 | 0.012 | 38.76 | 0.00007 | 4.80E-10 | 0.229 | 178726 |
| rs144968142 | C | G | -0.169 | 0.027 | 38.01 | 0.00002 | 7.04E-10 | 0.037 | 178726 |
| rs16986953 | A | G | 0.066 | 0.010 | 42.01 | 0.00012 | 9.08E-11 | 0.392 | 178726 |
| rs2107595 | A | G | 0.065 | 0.010 | 39.60 | 0.00011 | 3.11E-10 | 0.347 | 178726 |
| rs2184103 | G | A | 0.055 | 0.010 | 31.61 | 0.00008 | 1.88E-08 | 0.483 | 178726 |
| rs2327429 | C | T | -0.102 | 0.010 | 108.99 | 0.00029 | 1.63E-25 | 0.517 | 178726 |
| rs2519093 | T | C | 0.066 | 0.011 | 36.73 | 0.00008 | 1.35E-09 | 0.275 | 178726 |
| rs2677737 | T | C | -0.081 | 0.012 | 43.43 | 0.00008 | 4.40E-11 | 0.806 | 178726 |
| rs2738464 | C | G | 0.083 | 0.011 | 62.56 | 0.00014 | 2.59E-15 | 0.687 | 178726 |
| rs2839812 | A | T | -0.088 | 0.010 | 78.78 | 0.00021 | 6.93E-19 | 0.397 | 178726 |
| rs2869561 | C | T | -0.082 | 0.010 | 66.93 | 0.00019 | 2.81E-16 | 0.456 | 178726 |
| rs28709375 | G | A | 0.096 | 0.010 | 96.26 | 0.00026 | 1.01E-22 | 0.544 | 178726 |
| rs2886722 | G | A | 0.069 | 0.010 | 45.67 | 0.00012 | 1.40E-11 | 0.355 | 178726 |
| rs2892895 | A | G | -0.077 | 0.010 | 57.51 | 0.00015 | 3.36E-14 | 0.362 | 178726 |
| rs35752324 | T | C | -0.071 | 0.011 | 41.27 | 0.00009 | 1.32E-10 | 0.277 | 178726 |
| rs4432895 | T | C | 0.060 | 0.011 | 31.94 | 0.00007 | 1.59E-08 | 0.689 | 178726 |
| rs485838 | T | G | -0.058 | 0.010 | 31.88 | 0.00008 | 1.64E-08 | 0.334 | 178726 |
| rs4932463 | G | T | -0.089 | 0.010 | 82.42 | 0.00022 | 1.10E-19 | 0.544 | 178726 |
| rs57825321 | A | T | -0.089 | 0.015 | 36.23 | 0.00004 | 1.75E-09 | 0.124 | 178726 |
| rs60937209 | C | T | 0.067 | 0.010 | 46.44 | 0.00012 | 9.46E-12 | 0.540 | 178726 |
| rs651821 | T | C | -0.059 | 0.010 | 32.47 | 0.00009 | 1.21E-08 | 0.646 | 178726 |
| rs6694258 | A | C | -0.057 | 0.010 | 33.74 | 0.00009 | 6.29E-09 | 0.496 | 178726 |
| rs6704 | A | C | 0.065 | 0.012 | 31.13 | 0.00006 | 2.41E-08 | 0.230 | 178726 |
| rs6841473 | T | C | 0.094 | 0.011 | 79.51 | 0.00018 | 4.79E-19 | 0.314 | 178726 |
| rs7112216 | A | C | 0.064 | 0.010 | 43.08 | 0.00011 | 5.26E-11 | 0.462 | 178726 |
| rs73596816 | A | G | 0.183 | 0.026 | 48.45 | 0.00002 | 3.39E-12 | 0.042 | 178726 |
| rs75346744 | G | A | 0.195 | 0.020 | 92.79 | 0.00006 | 5.81E-22 | 0.062 | 178726 |
| rs769446 | C | T | -0.168 | 0.026 | 41.69 | 0.00002 | 1.07E-10 | 0.042 | 178726 |
| rs79717953 | T | C | 0.102 | 0.018 | 31.23 | 0.00003 | 2.29E-08 | 0.084 | 178726 |
| rs8076821 | C | T | 0.062 | 0.011 | 33.53 | 0.00007 | 7.02E-09 | 0.709 | 178726 |
| rs922131 | C | T | 0.062 | 0.010 | 37.25 | 0.00011 | 1.04E-09 | 0.560 | 178726 |
| rs9349379 | G | A | 0.138 | 0.011 | 150.96 | 0.00040 | 1.07E-34 | 0.652 | 178726 |
| rs9513112 | A | G | 0.074 | 0.010 | 54.47 | 0.00014 | 1.58E-13 | 0.382 | 178726 |
| rs9521686 | C | T | 0.074 | 0.011 | 49.01 | 0.00011 | 2.55E-12 | 0.691 | 178726 |
| rs9797885 | G | A | -0.066 | 0.010 | 45.46 | 0.00012 | 1.56E-11 | 0.484 | 178726 |
| **CHF** |  | | | | | | | | |
| rs11054389 | C | T | -0.146 | 0.032 | 21.09 | 0.00001 | 4.38E-06 | 0.059 | 178726 |
| rs11935601 | T | C | 0.068 | 0.015 | 20.01 | 0.00006 | 7.69E-06 | 0.582 | 178726 |
| rs139552246 | C | T | 0.216 | 0.047 | 21.11 | 0.00001 | 4.34E-06 | 0.031 | 178726 |
| rs142700595 | A | G | -0.240 | 0.053 | 20.53 | 0.00001 | 5.86E-06 | 0.023 | 178726 |
| rs16912489 | A | T | -0.138 | 0.029 | 22.56 | 0.00002 | 2.03E-06 | 0.072 | 178726 |
| rs17117025 | C | G | 0.272 | 0.060 | 20.85 | 0.00000 | 4.96E-06 | 0.016 | 178726 |
| rs1801270 | C | A | 0.069 | 0.015 | 22.23 | 0.00006 | 2.41E-06 | 0.477 | 178726 |
| rs255343 | G | A | -0.077 | 0.017 | 21.23 | 0.00004 | 4.07E-06 | 0.742 | 178726 |
| rs2609104 | G | A | -0.066 | 0.015 | 19.51 | 0.00005 | 1.00E-05 | 0.452 | 178726 |
| rs2742331 | A | T | -0.079 | 0.015 | 26.99 | 0.00007 | 2.05E-07 | 0.634 | 178726 |
| rs35429 | A | G | -0.096 | 0.017 | 30.79 | 0.00007 | 2.88E-08 | 0.257 | 178726 |
| rs35937380 | G | A | 0.081 | 0.018 | 20.54 | 0.00004 | 5.84E-06 | 0.217 | 178726 |
| rs3824359 | T | C | 0.075 | 0.017 | 20.15 | 0.00004 | 7.16E-06 | 0.265 | 178726 |
| rs4133201 | T | A | -0.136 | 0.030 | 20.12 | 0.00001 | 7.29E-06 | 0.938 | 178726 |
| rs4923918 | G | A | 0.095 | 0.021 | 21.18 | 0.00003 | 4.19E-06 | 0.147 | 178726 |
| rs6926631 | A | G | -0.098 | 0.022 | 19.75 | 0.00002 | 8.81E-06 | 0.872 | 178726 |
| rs72852791 | T | C | 0.110 | 0.024 | 20.86 | 0.00002 | 4.94E-06 | 0.106 | 178726 |
| rs72915796 | C | T | -0.223 | 0.045 | 24.13 | 0.00001 | 8.98E-07 | 0.033 | 178726 |
| rs7507813 | C | T | 0.128 | 0.028 | 20.35 | 0.00002 | 6.46E-06 | 0.073 | 178726 |
| rs76606350 | A | G | -0.230 | 0.049 | 22.41 | 0.00001 | 2.21E-06 | 0.028 | 178726 |
| rs78229461 | C | T | 0.076 | 0.015 | 26.85 | 0.00007 | 2.20E-07 | 0.454 | 178726 |
| rs9546526 | C | T | -0.108 | 0.021 | 25.95 | 0.00004 | 3.50E-07 | 0.139 | 178726 |
| **MI** |  | | | | | | | | |
| rs1054111 | C | A | -0.076 | 0.014 | 31.39 | 0.00009 | 2.11E-08 | 0.415 | 161206 |
| rs10743091 | C | T | 0.076 | 0.013 | 32.31 | 0.00011 | 1.31E-08 | 0.470 | 161206 |
| rs10818579 | G | A | 0.092 | 0.016 | 32.65 | 0.00007 | 1.10E-08 | 0.227 | 161206 |
| rs11105377 | C | T | -0.085 | 0.014 | 37.61 | 0.00011 | 8.66E-10 | 0.626 | 161206 |
| rs11739657 | T | A | -0.095 | 0.016 | 34.68 | 0.00008 | 3.89E-09 | 0.232 | 161206 |
| rs117598591 | C | A | 0.242 | 0.026 | 85.33 | 0.00007 | 2.53E-20 | 0.071 | 161206 |
| rs11925504 | G | A | -0.095 | 0.017 | 31.27 | 0.00006 | 2.25E-08 | 0.212 | 161206 |
| rs12225141 | C | T | 0.087 | 0.014 | 40.46 | 0.00012 | 2.00E-10 | 0.418 | 161206 |
| rs12589575 | G | T | -0.092 | 0.015 | 38.10 | 0.00010 | 6.71E-10 | 0.312 | 161206 |
| rs151193009 | C | T | -0.435 | 0.071 | 37.47 | 0.00000 | 9.28E-10 | 0.010 | 161206 |
| rs17612742 | T | C | 0.092 | 0.014 | 40.97 | 0.00012 | 1.54E-10 | 0.313 | 161206 |
| rs1870635 | C | T | 0.085 | 0.015 | 30.65 | 0.00008 | 3.09E-08 | 0.737 | 161206 |
| rs2246949 | C | T | 0.094 | 0.014 | 43.58 | 0.00012 | 4.08E-11 | 0.328 | 161206 |
| rs2327429 | T | C | -0.101 | 0.013 | 57.28 | 0.00019 | 3.78E-14 | 0.520 | 161206 |
| rs2519093 | C | T | 0.100 | 0.015 | 44.71 | 0.00011 | 2.28E-11 | 0.275 | 161206 |
| rs2677737 | C | T | -0.100 | 0.017 | 34.97 | 0.00007 | 3.35E-09 | 0.806 | 161206 |
| rs2869561 | T | C | -0.083 | 0.014 | 36.61 | 0.00011 | 1.45E-09 | 0.457 | 161206 |
| rs28709375 | A | G | 0.132 | 0.013 | 96.73 | 0.00032 | 7.96E-23 | 0.543 | 161206 |
| rs2886722 | A | G | 0.082 | 0.014 | 34.60 | 0.00010 | 4.05E-09 | 0.354 | 161206 |
| rs2891168 | A | G | 0.205 | 0.013 | 234.22 | 0.00077 | 7.16E-53 | 0.472 | 161206 |
| rs4762452 | A | C | -0.099 | 0.016 | 40.01 | 0.00009 | 2.53E-10 | 0.244 | 161206 |
| rs4932463 | T | G | -0.098 | 0.014 | 52.55 | 0.00015 | 4.18E-13 | 0.546 | 161206 |
| rs56171536 | A | C | 0.168 | 0.031 | 30.26 | 0.00002 | 3.77E-08 | 0.051 | 161206 |
| rs7118018 | T | G | -0.111 | 0.014 | 63.52 | 0.00018 | 1.59E-15 | 0.369 | 161206 |
| rs72789656 | C | G | 0.141 | 0.021 | 44.25 | 0.00006 | 2.89E-11 | 0.114 | 161206 |
| rs7412 | C | T | -0.206 | 0.034 | 35.94 | 0.00002 | 2.04E-09 | 0.042 | 161206 |
| rs76684042 | A | T | 0.273 | 0.031 | 79.51 | 0.00006 | 4.81E-19 | 0.067 | 161206 |
| rs781663 | A | G | 0.085 | 0.014 | 38.20 | 0.00011 | 6.38E-10 | 0.392 | 161206 |
| rs9349379 | A | G | 0.185 | 0.015 | 143.75 | 0.00043 | 4.04E-33 | 0.651 | 161206 |
| rs9513110 | T | G | 0.097 | 0.014 | 49.49 | 0.00014 | 1.99E-12 | 0.382 | 161206 |
| rs9521686 | T | C | 0.113 | 0.014 | 60.89 | 0.00017 | 6.03E-15 | 0.690 | 161206 |
| rs9797885 | A | G | -0.076 | 0.013 | 32.15 | 0.00011 | 1.43E-08 | 0.485 | 161206 |
| rs9897901 | C | T | 0.095 | 0.015 | 41.50 | 0.00010 | 1.18E-10 | 0.706 | 161206 |
| **SAP** |  | | | | | | | | |
| rs1054111 | A | C | -0.071 | 0.012 | 34.88 | 0.00010 | 3.50E-09 | 0.415 | 165047 |
| rs10757274 | G | A | 0.153 | 0.012 | 166.04 | 0.00049 | 5.41E-38 | 0.473 | 165047 |
| rs11107908 | T | C | -0.080 | 0.014 | 32.23 | 0.00007 | 1.37E-08 | 0.231 | 165047 |
| rs11191593 | C | T | -0.077 | 0.014 | 32.38 | 0.00007 | 1.27E-08 | 0.262 | 165047 |
| rs1144789 | G | A | -0.068 | 0.012 | 31.21 | 0.00009 | 2.32E-08 | 0.614 | 165047 |
| rs116873087 | C | G | 0.149 | 0.015 | 93.39 | 0.00022 | 4.29E-22 | 0.238 | 165047 |
| rs12225799 | G | C | 0.099 | 0.018 | 30.66 | 0.00004 | 3.07E-08 | 0.124 | 165047 |
| rs149856401 | G | C | 0.172 | 0.023 | 54.63 | 0.00004 | 1.46E-13 | 0.070 | 165047 |
| rs1862706 | A | C | 0.070 | 0.012 | 34.68 | 0.00010 | 3.89E-09 | 0.540 | 165047 |
| rs2327429 | C | T | -0.096 | 0.012 | 65.42 | 0.00019 | 6.05E-16 | 0.519 | 165047 |
| rs35303331 | G | A | -0.071 | 0.012 | 32.45 | 0.00010 | 1.22E-08 | 0.362 | 165047 |
| rs3748626 | G | T | 0.076 | 0.012 | 41.36 | 0.00012 | 1.27E-10 | 0.542 | 165047 |
| rs3751395 | A | C | -0.072 | 0.013 | 31.29 | 0.00008 | 2.22E-08 | 0.694 | 165047 |
| rs57301765 | A | G | 0.069 | 0.013 | 30.24 | 0.00008 | 3.82E-08 | 0.347 | 165047 |
| rs61904693 | C | T | -0.085 | 0.012 | 47.24 | 0.00014 | 6.28E-12 | 0.356 | 165047 |
| rs6496549 | C | T | -0.102 | 0.013 | 64.07 | 0.00019 | 1.20E-15 | 0.541 | 165047 |
| rs6841473 | T | C | 0.090 | 0.013 | 49.51 | 0.00012 | 1.97E-12 | 0.313 | 165047 |
| rs73596816 | A | G | 0.183 | 0.032 | 32.45 | 0.00002 | 1.22E-08 | 0.041 | 165047 |
| rs75346744 | G | A | 0.158 | 0.025 | 40.46 | 0.00003 | 2.00E-10 | 0.061 | 165047 |
| rs8027972 | C | T | -0.081 | 0.012 | 44.39 | 0.00013 | 2.70E-11 | 0.398 | 165047 |
| rs8182016 | C | G | -0.091 | 0.015 | 36.43 | 0.00007 | 1.59E-09 | 0.808 | 165047 |
| rs9349379 | G | A | 0.106 | 0.014 | 61.41 | 0.00016 | 4.64E-15 | 0.650 | 165047 |
| rs9797885 | G | A | -0.066 | 0.012 | 31.43 | 0.00009 | 2.07E-08 | 0.485 | 165047 |
| **UAP** |  | | | | | | | | |
| rs10048111 | C | T | 0.120 | 0.021 | 33.46 | 0.00009 | 7.29E-09 | 0.323 | 152105 |
| rs11226029 | A | G | -0.117 | 0.020 | 34.50 | 0.00011 | 4.26E-09 | 0.389 | 152105 |
| rs1131500 | T | C | -0.108 | 0.020 | 29.90 | 0.00009 | 4.56E-08 | 0.589 | 152105 |
| rs2869561 | C | T | -0.113 | 0.020 | 32.31 | 0.00010 | 1.32E-08 | 0.458 | 152105 |
| rs4977574 | G | A | 0.196 | 0.019 | 100.84 | 0.00035 | 9.98E-24 | 0.470 | 152105 |
| rs6842241 | A | C | 0.139 | 0.021 | 43.53 | 0.00012 | 4.18E-11 | 0.310 | 152105 |
| rs73596816 | A | G | 0.291 | 0.053 | 29.72 | 0.00002 | 4.99E-08 | 0.041 | 152105 |
| rs9349379 | G | A | 0.136 | 0.022 | 36.91 | 0.00011 | 1.24E-09 | 0.649 | 152105 |

Notes: CAD, coronary artery disease; MI, myocardial infarction; CHF, chronic heart failure; UAP, unstable angina pectoris; SAP, stable angina pectoris. MR analyses between the CADs and the risks of snoring traits, CADs and BMI were performed. For each of the CAD exposure, analysis of the outcome (snoring, habitual snoring, BMI) with the most genetic variants were shown in the table. *F* statistics for each SNP was calculated by (*β*/SE)^2^. We derived R^2^ of each genetic instrument for snoring using the following formula: R^2^=2 * MAF * (1-MAF) * (*β*/SD)^2^. *β* value was derived from the GWAS of snoring, in which we applied the BOLT-LMM software. The effect size of each SNP (*β*) on the quantitative scale, representing the effect of dosage on the genetic liability of CAD, could be converted to the traditional odds ratio using: OR = *β* / (μ * (1 - μ)), where μ = case fraction. SD meant the standard deviation of the effect size, calculated by the standard error of effect size (SE) * $\sqrt{sample size of GWAS of snoring}$. MAF meant the minor allele frequency of the corresponding SNP.

**Table S7. SVMR analysis for snoring on CADs applying genetic instruments at suggestively significant level (P<1E-05).**

| **Outcome** | **Method** | **No. of SNPs** | **OR** | **OR_LowerCI** | **OR_UpperCI** | **P** |
| --- | --- | --- | --- | --- | --- | --- |
| **Snoring** | | | | | | |
| CAD | Inverse variance weighted | 23 | 1.03 | 0.99 | 1.07 | 0.103 |
| CAD | Weighted median | 23 | 1.03 | 0.97 | 1.08 | 0.331 |
| CAD | MR Egger | 23 | 1.06 | 0.94 | 1.19 | 0.363 |
| CAD | RAPS | 23 | 1.04 | 1.01 | 1.08 | 0.025 |
| MI | Inverse variance weighted | 25 | 1.03 | 0.97 | 1.10 | 0.278 |
| MI | Weighted median | 25 | 1.00 | 0.93 | 1.08 | 0.943 |
| MI | MR Egger | 25 | 1.09 | 0.89 | 1.32 | 0.408 |
| MI | RAPS | 25 | 1.05 | 1.00 | 1.10 | 0.067 |
| CHF | Inverse variance weighted | 25 | 1.03 | 0.97 | 1.08 | 0.314 |
| CHF | Weighted median | 25 | 1.02 | 0.95 | 1.10 | 0.520 |
| CHF | MR Egger | 25 | 1.16 | 0.98 | 1.38 | 0.107 |
| CHF | RAPS | 25 | 1.04 | 0.98 | 1.09 | 0.204 |
| Angina | Inverse variance weighted | 25 | 1.00 | 0.94 | 1.06 | 0.970 |
| Angina | Weighted median | 25 | 1.01 | 0.94 | 1.08 | 0.760 |
| Angina | MR Egger | 25 | 0.97 | 0.80 | 1.18 | 0.792 |
| Angina | RAPS | 25 | 1.01 | 0.96 | 1.06 | 0.755 |
| SAP | Inverse variance weighted | 25 | 1.01 | 0.97 | 1.06 | 0.625 |
| SAP | Weighted median | 25 | 1.01 | 0.95 | 1.07 | 0.810 |
| SAP | MR Egger | 25 | 0.97 | 0.83 | 1.12 | 0.664 |
| SAP | RAPS | 25 | 1.02 | 0.98 | 1.07 | 0.317 |
| UAP | Inverse variance weighted | 25 | 1.04 | 0.97 | 1.11 | 0.311 |
| UAP | Weighted median | 25 | 1.04 | 0.94 | 1.14 | 0.470 |
| UAP | MR Egger | 25 | 1.21 | 0.96 | 1.52 | 0.113 |
| UAP | RAPS | 25 | 1.06 | 0.98 | 1.14 | 0.139 |
| **Habitual snoring** | | | | | | |
| CAD | Inverse variance weighted | 29 | 1.04 | 1.01 | 1.07 | 0.011 |
| CAD | Weighted median | 29 | 1.03 | 0.99 | 1.07 | 0.102 |
| CAD | MR Egger | 29 | 1.04 | 0.95 | 1.13 | 0.406 |
| CAD | RAPS | 29 | 1.04 | 1.01 | 1.07 | 0.007 |
| MI | Inverse variance weighted | 32 | 1.04 | 1.00 | 1.09 | 0.043 |
| MI | Weighted median | 32 | 1.03 | 0.98 | 1.09 | 0.231 |
| MI | MR Egger | 32 | 1.07 | 0.94 | 1.22 | 0.298 |
| MI | RAPS | 32 | 1.05 | 1.01 | 1.09 | 0.010 |
| CHF | Inverse variance weighted | 32 | 1.03 | 0.99 | 1.07 | 0.093 |
| CHF | Weighted median | 32 | 1.04 | 0.98 | 1.10 | 0.174 |
| CHF | MR Egger | 32 | 1.07 | 0.95 | 1.20 | 0.262 |
| CHF | RAPS | 32 | 1.03 | 0.99 | 1.08 | 0.088 |
| Angina | Inverse variance weighted | 32 | 1.03 | 0.99 | 1.07 | 0.157 |
| Angina | Weighted median | 32 | 1.01 | 0.96 | 1.06 | 0.800 |
| Angina | MR Egger | 32 | 1.00 | 0.88 | 1.13 | 0.970 |
| Angina | RAPS | 32 | 1.03 | 1.00 | 1.07 | 0.066 |
| SAP | Inverse variance weighted | 32 | 1.03 | 1.00 | 1.07 | 0.038 |
| SAP | Weighted median | 32 | 1.02 | 0.97 | 1.07 | 0.414 |
| SAP | MR Egger | 32 | 1.01 | 0.92 | 1.11 | 0.780 |
| SAP | RAPS | 32 | 1.04 | 1.01 | 1.07 | 0.021 |
| UAP | Inverse variance weighted | 32 | 1.08 | 1.03 | 1.14 | 0.002 |
| UAP | Weighted median | 32 | 1.07 | 0.99 | 1.14 | 0.085 |
| UAP | MR Egger | 32 | 1.12 | 0.96 | 1.31 | 0.164 |
| UAP | RAPS | 32 | 1.09 | 1.03 | 1.15 | 0.001 |

Notes: CAD, coronary artery disease; MI, myocardial infarction; CHF, chronic heart failure; UAP, unstable angina pectoris; SAP, stable angina pectoris; No. of SNPs, the number of single nucleotide polymorphisms selected into the two-sample MR analysis. SVMR, single variable MR. Odds ratio was applied as the coefficient, which expressed per 0.5-fold increase in the probability of CAD on the risks of outcomes.

**Table S8. Reverse SVMR analysis for CADs on BMI and snoring traits.**

| **Exposure** | **Methods** | **No. of SNPs** | **Coef** | **Coef _LowerCI** | **Coef _UpperCI** | **P** |
| --- | --- | --- | --- | --- | --- | --- |
| **Snoring** | | | | | | |
| Angina | Inverse variance weighted | 15 | 1.00 | 0.98 | 1.02 | 0.983 |
|  | Weighted median | 15 | 1.00 | 0.97 | 1.03 | 0.888 |
|  | MR Egger | 15 | 1.03 | 0.94 | 1.12 | 0.566 |
|  | RAPS | 15 | 1.00 | 0.98 | 1.02 | 0.981 |
| CAD | Inverse variance weighted | 48 | 1.00 | 0.98 | 1.01 | 0.685 |
|  | Weighted median | 48 | 0.99 | 0.97 | 1.01 | 0.538 |
|  | MR Egger | 48 | 0.98 | 0.94 | 1.03 | 0.452 |
|  | RAPS | 48 | 1.00 | 0.98 | 1.01 | 0.641 |
| CHF | Inverse variance weighted | 22 | 1.00 | 0.98 | 1.02 | 0.889 |
|  | Weighted median | 22 | 1.00 | 0.97 | 1.03 | 0.908 |
|  | MR Egger | 22 | 1.01 | 0.96 | 1.06 | 0.698 |
|  | RAPS | 22 | 1.00 | 0.98 | 1.02 | 0.892 |
| MI | Inverse variance weighted | 32 | 1.00 | 0.99 | 1.02 | 0.829 |
|  | Weighted median | 32 | 1.00 | 0.99 | 1.02 | 0.624 |
|  | MR Egger | 32 | 0.97 | 0.93 | 1.01 | 0.165 |
|  | RAPS | 32 | 1.00 | 0.99 | 1.01 | 0.813 |
| SAP | Inverse variance weighted | 23 | 0.99 | 0.97 | 1.01 | 0.461 |
|  | Weighted median | 23 | 0.99 | 0.96 | 1.02 | 0.387 |
|  | MR Egger | 23 | 1.02 | 0.94 | 1.10 | 0.635 |
|  | RAPS | 23 | 0.99 | 0.97 | 1.01 | 0.368 |
| UAP | Inverse variance weighted | 7 | 0.99 | 0.96 | 1.01 | 0.204 |
|  | Weighted median | 7 | 0.99 | 0.96 | 1.02 | 0.464 |
|  | MR Egger | 7 | 1.06 | 0.97 | 1.16 | 0.284 |
|  | RAPS | 7 | 0.99 | 0.96 | 1.01 | 0.212 |
| **Habitual snoring** | | | | | | |
| Angina | Inverse variance weighted | 15 | 0.99 | 0.96 | 1.02 | 0.652 |
|  | Weighted median | 15 | 1.02 | 0.99 | 1.06 | 0.242 |
|  | MR Egger | 15 | 0.99 | 0.87 | 1.12 | 0.839 |
|  | RAPS | 15 | 0.99 | 0.97 | 1.02 | 0.582 |
| CAD | Inverse variance weighted | 48 | 0.99 | 0.97 | 1.01 | 0.351 |
|  | Weighted median | 48 | 0.99 | 0.96 | 1.01 | 0.293 |
|  | MR Egger | 48 | 0.97 | 0.92 | 1.02 | 0.289 |
|  | RAPS | 48 | 0.99 | 0.97 | 1.01 | 0.250 |
| CHF | Inverse variance weighted | 22 | 1.00 | 0.97 | 1.02 | 0.863 |
|  | Weighted median | 22 | 0.99 | 0.95 | 1.03 | 0.593 |
|  | MR Egger | 22 | 1.00 | 0.93 | 1.07 | 0.970 |
|  | RAPS | 22 | 1.00 | 0.97 | 1.02 | 0.851 |
| MI | Inverse variance weighted | 31 | 0.99 | 0.97 | 1.00 | 0.113 |
|  | Weighted median | 31 | 0.98 | 0.95 | 1.00 | 0.071 |
|  | MR Egger | 31 | 0.98 | 0.93 | 1.03 | 0.401 |
|  | RAPS | 31 | 0.99 | 0.97 | 1.00 | 0.102 |
| SAP | Inverse variance weighted | 19 | 0.98 | 0.95 | 1.00 | 0.075 |
|  | Weighted median | 19 | 0.96 | 0.93 | 1.00 | 0.052 |
|  | MR Egger | 19 | 1.05 | 0.93 | 1.19 | 0.407 |
|  | RAPS | 19 | 0.97 | 0.95 | 1.00 | 0.077 |
| UAP | Inverse variance weighted | 5 | 0.98 | 0.94 | 1.01 | 0.165 |
|  | Weighted median | 5 | 0.97 | 0.93 | 1.01 | 0.114 |
|  | MR Egger | 5 | 1.03 | 0.71 | 1.49 | 0.883 |
|  | RAPS | 5 | 0.98 | 0.94 | 1.01 | 0.182 |
| **BMI** | | | | | | |
| Angina | Inverse variance weighted | 12 | -0.0007 | -0.0065 | 0.0052 | 0.827 |
|  | Weighted median | 12 | -0.0001 | -0.0073 | 0.0071 | 0.977 |
|  | MR Egger | 12 | 0.0083 | -0.0164 | 0.0330 | 0.525 |
|  | RAPS | 12 | -0.0007 | -0.0059 | 0.0045 | 0.801 |
| CAD | Inverse variance weighted | 45 | -0.0016 | -0.0048 | 0.0015 | 0.309 |
|  | Weighted median | 45 | -0.0006 | -0.0056 | 0.0043 | 0.806 |
|  | MR Egger | 45 | 0.0064 | -0.0033 | 0.0160 | 0.202 |
|  | RAPS | 45 | -0.0017 | -0.0049 | 0.0016 | 0.312 |
| CHF | Inverse variance weighted | 22 | -0.0014 | -0.0067 | 0.0040 | 0.617 |
|  | Weighted median | 22 | -0.0012 | -0.0075 | 0.0051 | 0.719 |
|  | MR Egger | 22 | 0.0037 | -0.0103 | 0.0177 | 0.607 |
|  | RAPS | 22 | -0.0014 | -0.0060 | 0.0031 | 0.539 |
| MI | Inverse variance weighted | 33 | 0.0004 | -0.0024 | 0.0033 | 0.775 |
|  | Weighted median | 33 | 0.0005 | -0.0037 | 0.0048 | 0.805 |
|  | MR Egger | 33 | -0.0069 | -0.0150 | 0.0012 | 0.107 |
|  | RAPS | 33 | 0.0004 | -0.0024 | 0.0032 | 0.768 |
| SAP | Inverse variance weighted | 18 | -0.0041 | -0.0087 | 0.0006 | 0.087 |
|  | Weighted median | 18 | -0.0020 | -0.0085 | 0.0046 | 0.557 |
|  | MR Egger | 18 | 0.0097 | -0.0061 | 0.0256 | 0.245 |
|  | RAPS | 18 | -0.0041 | -0.0089 | 0.0007 | 0.093 |
| UAP | Inverse variance weighted | 8 | -0.0015 | -0.0059 | 0.0030 | 0.513 |
|  | Weighted median | 8 | -0.0004 | -0.0061 | 0.0053 | 0.898 |
|  | MR Egger | 8 | 0.0080 | -0.0075 | 0.0234 | 0.350 |
|  | RAPS | 8 | -0.0015 | -0.0059 | 0.0028 | 0.493 |

Notes: BMI, body mass index; CAD, coronary artery disease; MI, myocardial infarction; CHF, chronic heart failure; UAP, unstable angina pectoris; SAP, stable angina pectoris; No. of SNPs, the number of single nucleotide polymorphisms selected into the two-sample MR analysis. SVMR, single variable MR.

For snoring traits as the outcomes, odds ratio was applied as the coefficient, which expressed per 0.5-fold increase in the probability of CAD on the risks of outcomes. For BMI as the outcome, *β* was applied as the coefficients, which expressed per 0.5-fold increase in the probability of CAD on the levels of BMI.

**Table S9. MVMR analysis for snoring, BMI on CADs using snoring loci at suggestively significant level (P<1E-05).**

| **Exposure** | **Outcome** | **Methods** | **OR** | **OR_LowerCI** | **OR_UpperCI** | **P** |
| --- | --- | --- | --- | --- | --- | --- |
| Snoring | MI | MVMR-IVW | 1.03 | 0.97 | 1.09 | 0.30348708 |
| BMI | MI | MVMR-IVW | 1.36 | 1.17 | 1.58 | 7.00E-05 |
| Snoring | MI | MVMR-Egger | 1.01 | 0.93 | 1.10 | 0.83310483 |
| BMI | MI | MVMR-Egger | 1.32 | 1.12 | 1.56 | 0.00106882 |
| Snoring | CHF | MVMR-IVW | 0.99 | 0.93 | 1.05 | 0.64181054 |
| BMI | CHF | MVMR-IVW | 1.50 | 1.29 | 1.74 | 1.09E-07 |
| Snoring | CHF | MVMR-Egger | 0.99 | 0.91 | 1.08 | 0.85531794 |
| BMI | CHF | MVMR-Egger | 1.51 | 1.28 | 1.79 | 1.21E-06 |
| Snoring | Angina | MVMR-IVW | 1.00 | 0.94 | 1.06 | 0.95617704 |
| BMI | Angina | MVMR-IVW | 1.25 | 1.08 | 1.45 | 0.00278587 |
| Snoring | Angina | MVMR-Egger | 0.98 | 0.90 | 1.06 | 0.55777697 |
| BMI | Angina | MVMR-Egger | 1.22 | 1.03 | 1.44 | 0.01811262 |
| Snoring | SAP | MVMR-IVW | 1.02 | 0.97 | 1.08 | 0.43821412 |
| BMI | SAP | MVMR-IVW | 1.19 | 1.04 | 1.37 | 0.00958085 |
| Snoring | SAP | MVMR-Egger | 0.99 | 0.92 | 1.07 | 0.85814511 |
| BMI | SAP | MVMR-Egger | 1.16 | 1.00 | 1.34 | 0.05688508 |
| Snoring | UAP | MVMR-IVW | 1.04 | 0.96 | 1.13 | 0.32811591 |
| BMI | UAP | MVMR-IVW | 1.25 | 1.01 | 1.55 | 0.03984166 |
| Snoring | UAP | MVMR-Egger | 1.04 | 0.93 | 1.18 | 0.48262398 |
| BMI | UAP | MVMR-Egger | 1.25 | 0.99 | 1.59 | 0.06356475 |
| Snoring | CAD | MVMR-IVW | 1.04 | 0.99 | 1.09 | 0.10739507 |
| BMI | CAD | MVMR-IVW | 1.23 | 1.10 | 1.39 | 0.00043731 |
| Snoring | CAD | MVMR-Egger | 1.02 | 0.96 | 1.09 | 0.49732282 |
| BMI | CAD | MVMR-Egger | 1.21 | 1.06 | 1.38 | 0.00379385 |
| Habitual | MI | MVMR-IVW | 1.04 | 1.00 | 1.08 | 0.06188767 |
| BMI | MI | MVMR-IVW | 1.33 | 1.15 | 1.54 | 0.00015113 |
| Habitual | MI | MVMR-Egger | 0.99 | 0.94 | 1.06 | 0.83700274 |
| BMI | MI | MVMR-Egger | 1.25 | 1.07 | 1.46 | 0.00431401 |
| Habitual | CHF | MVMR-IVW | 1.01 | 0.97 | 1.05 | 0.76013957 |
| BMI | CHF | MVMR-IVW | 1.50 | 1.29 | 1.73 | 4.22E-08 |
| Habitual | CHF | MVMR-Egger | 1.00 | 0.94 | 1.07 | 0.92266131 |
| BMI | CHF | MVMR-Egger | 1.49 | 1.27 | 1.74 | 5.24E-07 |
| Habitual | Angina | MVMR-IVW | 1.03 | 0.99 | 1.07 | 0.16961931 |
| BMI | Angina | MVMR-IVW | 1.18 | 1.03 | 1.37 | 0.02072778 |
| Habitual | Angina | MVMR-Egger | 1.00 | 0.95 | 1.07 | 0.86878499 |
| BMI | Angina | MVMR-Egger | 1.15 | 0.99 | 1.34 | 0.076195 |
| Habitual | SAP | MVMR-IVW | 1.04 | 1.00 | 1.08 | 0.03269704 |
| BMI | SAP | MVMR-IVW | 1.14 | 1.00 | 1.29 | 0.04742274 |
| Habitual | SAP | MVMR-Egger | 1.02 | 0.97 | 1.08 | 0.37183968 |
| BMI | SAP | MVMR-Egger | 1.11 | 0.97 | 1.28 | 0.1172573 |
| Habitual | UAP | MVMR-IVW | 1.09 | 1.02 | 1.15 | 0.00617337 |
| BMI | UAP | MVMR-IVW | 1.16 | 0.94 | 1.43 | 0.1672142 |
| Habitual | UAP | MVMR-Egger | 1.07 | 0.98 | 1.17 | 0.1343001 |
| BMI | UAP | MVMR-Egger | 1.14 | 0.91 | 1.43 | 0.26920098 |
| Habitual | CAD | MVMR-IVW | 1.04 | 1.01 | 1.08 | 0.00997947 |
| BMI | CAD | MVMR-IVW | 1.20 | 1.07 | 1.35 | 0.00182625 |
| Habitual | CAD | MVMR-Egger | 1.02 | 0.97 | 1.07 | 0.36703519 |
| BMI | CAD | MVMR-Egger | 1.17 | 1.03 | 1.33 | 0.01232354 |

Notes: BMI, body mass index; CAD, coronary artery disease; MI, myocardial infarction; CHF, chronic heart failure; UAP, unstable angina pectoris; SAP, stable angina pectoris; MVMR, multivariable MR; IVW, inverse variance weighted. For snoring, estimates were expressed per 0.5-fold increase in the probability of snoring (MVMR adjusted for BMI) on the risk of CADs. For BMI, estimates were expressed per 1 SD increase in the BMI (MVMR adjusted for snoring) on the risk of CADs.

**Table S10. Reverse MVMR analysis for CADs, BMI on the risks of snoring traits.**

| **Exposure** | **Methods** | **OR** | **OR_LowerCI** | **OR_UpperCI** | **P** |
| --- | --- | --- | --- | --- | --- |
| **Snoring** | | | | | |
| MI | MVMR-IVW | 1.01 | 1.00 | 1.03 | 0.09887769 |
| BMI | MVMR-IVW | 1.47 | 1.32 | 1.63 | 4.78E-13 |
| MI | MVMR-Egger | 1.01 | 0.98 | 1.03 | 0.6391986 |
| BMI | MVMR-Egger | 1.43 | 1.28 | 1.61 | 1.24E-09 |
| CHF | MVMR-IVW | 0.99 | 0.96 | 1.02 | 0.47570986 |
| BMI | MVMR-IVW | 1.48 | 1.34 | 1.63 | 2.21E-15 |
| CHF | MVMR-Egger | 1.00 | 0.97 | 1.04 | 0.93585311 |
| BMI | MVMR-Egger | 1.52 | 1.36 | 1.70 | 8.60E-14 |
| Angina | MVMR-IVW | 1.03 | 1.00 | 1.06 | 0.07840954 |
| BMI | MVMR-IVW | 1.45 | 1.32 | 1.60 | 8.71E-14 |
| Angina | MVMR-Egger | 1.02 | 0.99 | 1.06 | 0.23811584 |
| BMI | MVMR-Egger | 1.45 | 1.31 | 1.60 | 1.20E-12 |
| SAP | MVMR-IVW | 1.02 | 1.00 | 1.05 | 0.11050735 |
| BMI | MVMR-IVW | 1.47 | 1.32 | 1.63 | 3.78E-13 |
| SAP | MVMR-Egger | 1.01 | 0.97 | 1.04 | 0.61732401 |
| BMI | MVMR-Egger | 1.45 | 1.31 | 1.62 | 2.29E-12 |
| UAP | MVMR-IVW | 1.02 | 0.99 | 1.04 | 0.1864691 |
| BMI | MVMR-IVW | 1.47 | 1.33 | 1.62 | 6.23E-14 |
| UAP | MVMR-Egger | 1.01 | 0.98 | 1.04 | 0.54573777 |
| BMI | MVMR-Egger | 1.46 | 1.32 | 1.62 | 3.70E-13 |
| CAD | MVMR-IVW | 1.02 | 0.99 | 1.04 | 0.13793873 |
| BMI | MVMR-IVW | 1.48 | 1.33 | 1.65 | 2.85E-12 |
| CAD | MVMR-Egger | 1.02 | 0.99 | 1.05 | 0.23023977 |
| BMI | MVMR-Egger | 1.48 | 1.32 | 1.67 | 3.83E-11 |
| **Habitual snoring** | | | | | |
| MI | MVMR-IVW | 1.01 | 0.99 | 1.03 | 0.26258265 |
| BMI | MVMR-IVW | 1.69 | 1.48 | 1.93 | 2.39E-14 |
| MI | MVMR-Egger | 0.99 | 0.96 | 1.02 | 0.56334013 |
| BMI | MVMR-Egger | 1.58 | 1.37 | 1.84 | 8.43E-10 |
| CHF | MVMR-IVW | 0.98 | 0.95 | 1.01 | 0.23930176 |
| BMI | MVMR-IVW | 1.71 | 1.50 | 1.94 | 3.06E-16 |
| CHF | MVMR-Egger | 0.99 | 0.95 | 1.04 | 0.68680172 |
| BMI | MVMR-Egger | 1.76 | 1.52 | 2.04 | 4.12E-14 |
| Angina | MVMR-IVW | 1.03 | 0.99 | 1.06 | 0.16466516 |
| BMI | MVMR-IVW | 1.66 | 1.46 | 1.88 | 3.09E-15 |
| Angina | MVMR-Egger | 1.02 | 0.97 | 1.07 | 0.38571672 |
| BMI | MVMR-Egger | 1.65 | 1.45 | 1.88 | 5.88E-14 |
| SAP | MVMR-IVW | 1.02 | 0.98 | 1.05 | 0.33086171 |
| BMI | MVMR-IVW | 1.69 | 1.48 | 1.94 | 1.95E-14 |
| SAP | MVMR-Egger | 1.00 | 0.96 | 1.05 | 0.87665678 |
| BMI | MVMR-Egger | 1.67 | 1.46 | 1.92 | 1.31E-13 |
| UAP | MVMR-IVW | 1.01 | 0.98 | 1.04 | 0.4105085 |
| BMI | MVMR-IVW | 1.68 | 1.47 | 1.92 | 1.79E-14 |
| UAP | MVMR-Egger | 1.00 | 0.96 | 1.04 | 0.97417493 |
| BMI | MVMR-Egger | 1.66 | 1.45 | 1.90 | 1.25E-13 |
| CAD | MVMR-IVW | 1.01 | 0.99 | 1.04 | 0.30240501 |
| BMI | MVMR-IVW | 1.74 | 1.51 | 2.01 | 1.62E-14 |
| CAD | MVMR-Egger | 1.02 | 0.98 | 1.05 | 0.41148751 |
| BMI | MVMR-Egger | 1.75 | 1.50 | 2.03 | 4.69E-13 |

Notes: BMI, body mass index; CAD, coronary artery disease; MI, myocardial infarction; CHF, chronic heart failure; UAP, unstable angina pectoris; SAP, stable angina pectoris; MVMR, multivariable MR; IVW, inverse variance weighted. For CADs, estimates were expressed per 0.5-fold increase in the probability of the CAD (MVMR adjusted for BMI) on the risk of outcomes (snoring or habitual snoring). For BMI, estimates were expressed per 1 SD increase in the BMI (MVMR adjusted for CAD) on the risk of outcomes.

**Table S11. Tests for the single variable MR.**

| **Exposure** | **Outcome** | **Horizonal Pleiotropy test** | | |  | **Heterogeneity test** | |  | **MR Steiger test** | |
| --- | --- | --- | --- | --- | --- | --- | --- | --- | --- | --- |
|  |  | **Egger.inter** | **Egger.SE** | **P.inter** |  | ***Q*** | **P.heterogeneity** |  | **Correct causal directions** | **P.Steiger** |
| Snoring (P<5E-08) | CAD | -0.020 | 0.048 | 0.743 |  | 2.631 | 0.268 |  | TRUE | 5.95E-13 |
|  | MI | -0.042 | 0.055 | 0.589 |  | 2.504 | 0.286 |  | TRUE | 4.51E-10 |
|  | CHF | -0.108 | 0.048 | 0.266 |  | 5.06 | 0.08 |  | TRUE | 2.16E-09 |
|  | Angina | -0.039 | 0.054 | 0.604 |  | 2.34 | 0.31 |  | TRUE | 2.67E-11 |
|  | SAP | -0.012 | 0.039 | 0.807 |  | 0.659 | 0.719 |  | TRUE | 2.71E-13 |
|  | UAP | -0.078 | 0.064 | 0.439 |  | 1.508 | 0.471 |  | TRUE | 7.18E-12 |
| Habitual snoring (P<5E-08) | CAD | 0.029 | 0.053 | 0.687 |  | 3.383 | 0.184 |  | TRUE | 2.96E-12 |
|  | MI | -0.006 | 0.045 | 0.915 |  | 0.65 | 0.722 |  | TRUE | 1.01E-11 |
|  | CHF | -0.108 | 0.049 | 0.274 |  | 4.976 | 0.083 |  | TRUE | 3.88E-12 |
|  | Angina | 0.024 | 0.063 | 0.772 |  | 2.265 | 0.322 |  | TRUE | 1.78E-14 |
|  | SAP | 0.044 | 0.068 | 0.636 |  | 4.089 | 0.129 |  | TRUE | 2.43E-13 |
|  | UAP | -0.05 | 0.086 | 0.664 |  | 2.286 | 0.319 |  | TRUE | 8.09E-14 |
| Snoring (P<1E-05) | CAD | -0.004 | 0.008 | 0.660 |  | 22.085 | 0.455 |  | TRUE | 1.98E-61 |
|  | MI | -0.007 | 0.013 | 0.596 |  | 36.639 | 0.048 |  | TRUE | 3.87E-53 |
|  | CHF | -0.017 | 0.012 | 0.165 |  | 23.076 | 0.515 |  | TRUE | 5.27E-63 |
|  | Angina | 0.003 | 0.013 | 0.791 |  | 35.584 | 0.06 |  | TRUE | 2.19E-54 |
|  | SAP | 0.006 | 0.01 | 0.543 |  | 26.789 | 0.314 |  | TRUE | 1.24E-57 |
|  | UAP | -0.022 | 0.015 | 0.174 |  | 18.934 | 0.756 |  | TRUE | 1.46E-57 |
| Habitual snoring (P<1E-05) | CAD | -0.0002 | 0.007 | 0.979 |  | 31.831 | 0.281 |  | TRUE | 9.43E-85 |
|  | MI | -0.005 | 0.01 | 0.67 |  | 43.751 | 0.064 |  | TRUE | 4.00E-79 |
|  | CHF | -0.006 | 0.01 | 0.534 |  | 26.566 | 0.694 |  | TRUE | 3.43E-91 |
|  | Angina | 0.005 | 0.01 | 0.604 |  | 40.164 | 0.125 |  | TRUE | 1.72E-80 |
|  | SAP | 0.003 | 0.008 | 0.678 |  | 25.164 | 0.76 |  | TRUE | 1.52E-86 |
|  | UAP | -0.006 | 0.013 | 0.655 |  | 23.948 | 0.813 |  | TRUE | 7.49E-82 |
| BMI | CAD | 0.001 | 0.006 | 0.808 |  | 62.512 | 0.043 |  | TRUE | 1.78E-239 |
|  | MI | 0.004 | 0.007 | 0.581 |  | 65.083 | 0.105 |  | TRUE | 1.43E-261 |
|  | CHF | -0.013 | 0.007 | 0.064 |  | 57.015 | 0.294 |  | TRUE | 1.17E-280 |
|  | Angina | 0.001 | 0.006 | 0.854 |  | 58.173 | 0.291 |  | TRUE | 2.66E-278 |
|  | SAP | -0.003 | 0.006 | 0.565 |  | 60.714 | 0.191 |  | TRUE | 1.74E-279 |
|  | UAP | -0.003 | 0.011 | 0.785 |  | 75.375 | 0.023 |  | TRUE | 2.90E-265 |
| Angina | Snoring | -0.007 | 0.012 | 0.556 |  | 17.148 | 0.248 |  | TRUE | 3.29E-41 |
| CAD |  | 0.003 | 0.005 | 0.514 |  | 61.280 | 0.079 |  | TRUE | 1.16E-158 |
| CHF |  | -0.002 | 0.007 | 0.717 |  | 14.061 | 0.867 |  | TRUE | 8.08E-25 |
| MI |  | 0.01 | 0.006 | 0.127 |  | 36.916 | 0.214 |  | TRUE | 5.20E-102 |
| SAP |  | -0.006 | 0.009 | 0.479 |  | 32.041 | 0.077 |  | TRUE | 1.03E-58 |
| UAP |  | -0.023 | 0.015 | 0.176 |  | 4.463 | 0.614 |  | TRUE | 2.75E-15 |
| Angina | Habitual snoring | 0.002 | 0.016 | 0.922 |  | 20.153 | 0.125 |  | TRUE | 6.83E-31 |
| CAD |  | 0.005 | 0.006 | 0.436 |  | 69.894 | 0.017 |  | TRUE | 9.42E-118 |
| CHF |  | -0.001 | 0.009 | 0.912 |  | 24.373 | 0.275 |  | TRUE | 1.42E-15 |
| MI |  | 0.002 | 0.007 | 0.744 |  | 31.787 | 0.377 |  | TRUE | 3.47E-75 |
| SAP |  | -0.016 | 0.013 | 0.212 |  | 15.993 | 0.593 |  | TRUE | 3.42E-32 |
| UAP |  | -0.017 | 0.057 | 0.79 |  | 1.025 | 0.906 |  | TRUE | 2.59E-10 |
| Angina | BMI | -0.005 | 0.007 | 0.481 |  | 14.579 | 0.203 |  | TRUE | 3.69E-33 |
| CAD |  | -0.004 | 0.002 | 0.092 |  | 34.084 | 0.859 |  | TRUE | 4.62E-146 |
| CHF |  | -0.003 | 0.004 | 0.449 |  | 30.967 | 0.074 |  | TRUE | 7.92E-19 |
| MI |  | 0.005 | 0.003 | 0.071 |  | 34.281 | 0.359 |  | TRUE | 2.04E-103 |
| SAP |  | -0.008 | 0.004 | 0.093 |  | 12.02 | 0.799 |  | TRUE | 3.01E-49 |
| UAP |  | -0.008 | 0.006 | 0.258 |  | 7.708 | 0.359 |  | TRUE | 3.74E-21 |

Notes: BMI, body mass index; CAD, coronary artery disease; MI, myocardial infarction; CHF, chronic heart failure; UAP, unstable angina pectoris; SAP, stable angina pectoris; SVMR, single variable MR; MVMR, multivariable MR; IVW, inverse variance weighted; RAPS, robust adjusted profile score; MR-PRESSO, MR-Pleiotropy RESidual Sum and Outlier. Inverse variance weighted Cochran’s *Q* test was performed for the test of heterogeneity. MR-Egger regression was performed to test for the horizontal pleiotropy. MR Steiger analysis was applied to elucidate whether the assumption that the exposure causes the outcome was valid, testing whether the correlation of exposure SNPs in the outcomes was less than the correlation with exposure. (from the exposure to the outcome).

**Table S12. Tests for the multivariable MR.**

| **Exposure** | **Outcome** | **No. of SNPs** | **Heterogeneity test** | |  | **Horizonal Pleiotropy test** | | |  | **Variance inflation factors** |
| --- | --- | --- | --- | --- | --- | --- | --- | --- | --- | --- |
|  |  |  | ***Q*** | **P.Heterogeneity** |  | **Egger.inter** | **Egger.SE** | **P.inter** |  |  |
| Snoring (P<5E-08), BMI | MI | 55 | 63.859 | 0.146 |  | -0.001 | 0.004 | 0.896 |  | 2.031 |
|  | CHF | 55 | 56.114 | 0.359 |  | -3.10E-04 | 0.004 | 0.937 |  | 1.993 |
|  | Angina | 56 | 62.089 | 0.210 |  | 0.002 | 0.004 | 0.600 |  | 2.035 |
|  | SAP | 55 | 59.231 | 0.259 |  | 0.002 | 0.003 | 0.582 |  | 1.989 |
|  | UAP | 56 | 73.438 | 0.040 |  | 1.70E-04 | 0.006 | 0.977 |  | 2.033 |
|  | CAD | 55 | 56.555 | 0.344 |  | 0.001 | 0.003 | 0.764 |  | 1.940 |
| Habitual snoring (P<5E-08), BMI | MI | 55 | 62.269 | 0.180 |  | 0.007 | 0.004 | 0.079 |  | 2.122 |
|  | CHF | 55 | 56.171 | 0.357 |  | -0.001 | 0.004 | 0.875 |  | 2.071 |
|  | Angina | 56 | 58.615 | 0.310 |  | 0.003 | 0.004 | 0.387 |  | 2.120 |
|  | SAP | 55 | 58.836 | 0.270 |  | 0.001 | 0.003 | 0.842 |  | 2.073 |
|  | UAP | 56 | 74.261 | 0.035 |  | 0.003 | 0.006 | 0.661 |  | 2.118 |
|  | CAD | 55 | 55.763 | 0.371 |  | 0.001 | 0.003 | 0.705 |  | 2.064 |
| Snoring (P<1E-05), BMI | MI | 74 | 95.926 | 0.031 |  | 0.003 | 0.004 | 0.470 |  | 1.326 |
|  | CHF | 74 | 79.485 | 0.255 |  | -0.001 | 0.004 | 0.841 |  | 1.327 |
|  | Angina | 74 | 94.058 | 0.042 |  | 0.003 | 0.004 | 0.441 |  | 1.327 |
|  | SAP | 74 | 97.458 | 0.025 |  | 0.004 | 0.003 | 0.308 |  | 1.327 |
|  | UAP | 74 | 92.404 | 0.053 |  | 0.000 | 0.006 | 0.980 |  | 1.327 |
|  | CAD | 73 | 86.710 | 0.099 |  | 0.001 | 0.003 | 0.589 |  | 1.326 |
| Habitual snoring (P<1E-05), BMI | MI | 80 | 99.735 | 0.049 |  | 0.007 | 0.004 | 0.041 |  | 1.239 |
|  | CHF | 80 | 79.143 | 0.443 |  | 0.001 | 0.004 | 0.882 |  | 1.236 |
|  | Angina | 80 | 95.279 | 0.089 |  | 0.004 | 0.004 | 0.297 |  | 1.239 |
|  | SAP | 80 | 92.645 | 0.123 |  | 0.002 | 0.003 | 0.453 |  | 1.238 |
|  | UAP | 80 | 97.069 | 0.071 |  | 0.003 | 0.005 | 0.632 |  | 1.237 |
|  | CAD | 78 | 89.973 | 0.131 |  | 0.003 | 0.003 | 0.254 |  | 1.397 |
| MI, BMI | Snoring | 74 | 119.512 | 3.71E-04 |  | 0.002 | 0.002 | 0.338 |  | 1.004 |
| CHF, BMI |  | 64 | 89.859 | 0.012 |  | -0.002 | 0.002 | 0.293 |  | 1.004 |
| Angina, BMI |  | 59 | 84.470 | 0.011 |  | 0.001 | 0.002 | 0.742 |  | 1.200 |
| SAP, BMI |  | 61 | 95.935 | 0.002 |  | 0.003 | 0.002 | 0.224 |  | 1.079 |
| UAP, BMI |  | 56 | 85.827 | 0.004 |  | 0.001 | 0.002 | 0.520 |  | 1.055 |
| CAD, BMI |  | 74 | 121.007 | 2.67E-04 |  | -4.03E-04 | 0.002 | 0.858 |  | 1.018 |
| MI, BMI | Habitual snoring | 74 | 130.059 | 3.34E-05 |  | 0.006 | 0.003 | 0.054 |  | 1.004 |
| CHF, BMI |  | 64 | 102.891 | 0.001 |  | -0.003 | 0.003 | 0.392 |  | 1.004 |
| Angina, BMI |  | 59 | 91.579 | 0.002 |  | 0.001 | 0.003 | 0.715 |  | 1.200 |
| SAP, BMI |  | 61 | 105.533 | 1.89E-04 |  | 0.003 | 0.003 | 0.330 |  | 1.079 |
| UAP, BMI |  | 56 | 98.537 | 2.05E-04 |  | 0.003 | 0.003 | 0.344 |  | 1.055 |
| CAD, BMI |  | 74 | 131.948 | 2.12E-05 |  | -3.08E-04 | 0.003 | 0.916 |  | 1.018 |

Notes: BMI, body mass index; CAD, coronary artery disease; MI, myocardial infarction; CHF, chronic heart failure; UAP, unstable angina pectoris; SAP, stable angina pectoris; SVMR, single variable MR; MVMR, multivariable MR; IVW, inverse variance weighted; No. of SNPs, number of SNPs used in the multivariable MR analysis. Inverse variance weighted Cochran’s *Q* test was performed for the test of heterogeneity. MR-Egger regression was performed to test for the horizontal pleiotropy.

**References**

1. Burgess S, Thompson SG. Mendelian Randomization; Methods for Causal Inference Using Genetic Variants; Second Edition, 2021.
